# Rule-based modelling provides an extendable framework for comparing candidate mechanisms underpinning clathrin polymerisation

**DOI:** 10.1038/s41598-018-23829-x

**Published:** 2018-04-04

**Authors:** Anatoly Sorokin, Katharina F. Heil, J. Douglas Armstrong, Oksana Sorokina

**Affiliations:** 1 Institute of Cell Biophysics, RAS, Pushchino, 142290 Russia; 2 Moscow Institute of Physics and Technology, 141700 Dolgoprudnyi, Moscow Region Russia; 3 School of Informatics, University of Edinburgh, Edinburgh, EH8 9AB United Kingdom

## Abstract

Polymerisation of clathrin is a key process that underlies clathrin-mediated endocytosis. Clathrin-coated vesicles are responsible for cell internalization of external substances required for normal homeostasis and life –sustaining activity. There are several hypotheses describing formation of closed clathrin structures. According to one of the proposed mechanisms cage formation may start from a flat lattice buildup on the cellular membrane, which is later transformed into a curved structure. Creation of the curved surface requires rearrangement of the lattice, induced by additional molecular mechanisms. Different potential mechanisms require a modeling framework that can be easily modified to compare between them. We created an extendable rule-based model that describes polymerisation of clathrin molecules and various scenarios of cage formation. Using Global Sensitivity Analysis (GSA) we obtained parameter sets describing clathrin pentagon closure and the emergence/production and closure of large-size clathrin cages/vesicles. We were able to demonstrate that the model can reproduce budding of the clathrin cage from an initial flat array.

## Introduction

Clathrin is the major protein component of clathrin–mediated endocytosis (CME)^1,2^. Due to its particular shape and (auto-) polymerization capacity, clathrin is believed to induce the cell membrane to adopt a vesicular shape. A range of different mechanisms have been proposed for this process^3–5^, from a few minimalistic ones propose that clathrin polymerization alone is sufficient to generate buds in a planar membrane^6^ to the consensus that describe the orchestrated action of additional proteins and signaling cascades on the intracellular side of the membrane, so that ~30 proteins directly participate in the various steps of endocytosis^1,7–9^.

The structural properties of clathrin have been extensively investigated with respect to their role in vesicle formation. Usually a clathrin molecule is composed of one heavy (~190 kDa) as well as one light chain (~25 kD) and is about 475 Ångström (Å) in length^10^. Within the cell clathrin exists in a form of trimers (triskelia), consisting of three clathrin molecules (three heavy and three light chains respectively), where individual clathrin monomers are referred to as “legs”. Deviating from the normal 1:1 ratio between light and heavy chain several studies have also revealed the existence of triskelia with fewer light chains. Triskelia formation itself does not seem to be influenced by a loss of light chain molecules^11^, but regulatory control of vesicle formation and cargo selection have been proposed.

Due to its internal trimeric structure every single clathrin molecule in the triskelia complex can polymerize with another clathrin molecule from a different clathrin triskelia. Hence every triskelia is able to undergo interactions with three further triskelia. This leads to the formation of dimers and trimers, which can grow to construct large polymers. However, in a normal biological context, hexagonal and pentagonal shapes are among the most frequently observed^12,13^. Specific combinations of these shapes induce the formation of the typical vesicle closed spherical structure. Normally, closed structures contain 12 pentagonal faces and (N-20)/2 hexagonal faces. The fixed relative numbers between pentagonal and hexagonal faces are based on geometric constraints, given the clathrin structure and minimal flexibility of the trimer legs. Based on the number (N) of triskelia different sphere sizes can emerge, three of which are well defined: The mini-coat, hexagonal-barrel and soccer ball^13^.

Since its discovery in 1975^14^, significant attention has been focused on the mechanism of clathrin polymerisation. It was highlighted in^1^ that understanding CME is not possible without proper knowledge of its key process, the clathrin cage formation. Although it was experimentally shown that clathrin self-assembles following pH decrease from 8 to 6.5^15^ or under bivalent cation administration^16^, to obtain biologically realistic vesicle shapes the participation of external regulatory proteins is likely critical^1^.

A range of computational models for clathrin self-assembly exists that describes the formation of clathrin cages^12,13,17–19^, or pits and vesicles^13,15,20^. Early models considered the association of 3-valent polymers with equi-reactive binding sites from the Flori’s theory point of view with^20^ or without^21^ allowance for intramolecular loop formation. These studies dissected the dependence of the solution/gel phase transition linked to the critical concentration of the monomer on the equilibrium constants of different steps of the polymerisation process. In the early theoretical models of multivalent condensation, the term “gel” was used to describe the situation when the majority of agents participate in one global complex. There are two phases in such system: a solution consisting of many small complexes and monomers, and a gel, composed of one global complex and a few free monomers. The formation of the global complex is a key phase transition in the systems dynamics. Prior to gel formation, the dynamics of the system are driven by bi-molecular reactions (when two complexes form a bigger one, or a monomer attaches to the complex). After gel formation, the dynamics are driven by uni-molecular reactions within the complex. The key finding of Falk and Thomas^20^ is that before the transition to the gel phase, uni-molecular reactions are negligible.

In particular, it was shown by Pastan and Willingham^15^, that the critical concentration of clathrin, sufficient for the phase transition was 30 mg/ml. Taking into account that the triskelia molecular mass is about 640 kDa, this value corresponds to the molar concentration of 46 µM, or approximately 55000 triskelia per eukaryotic cell.

More recent studies examined the assembly of 5- and 6- member rings in parallel with investigation of how different physical triskelia characteristics might impact on cage formation. These characteristics include triskelia rigidity^21^, their asymmetry^17^, emergent tension during cage closure^22^ and the effects of superficial membrane tension^23^. These studies provide approximations of binding energy between the chains of the neighbouring clathin triskelia^17^.

The polymerisation process alone presents a significant challenge for mechanistic modeling, as the number of molecular species, which have to be described, grows exponentially with the number of available monomers. Rule-based modeling^24–26^ provides a viable solution allowing a network–free simulation technique^27–29^. It uses ‘lumped’ reaction rules to concisely represent molecule interactions. One can assume the rules as implicit combinations of different reactions into classes, where all the members of the same class perform a common transformation. This modeling approach is generally exploited for large-scale biochemical systems to overcome combinatorial complexity and it has previously demonstrated its effectiveness in simulations of ligand-receptor complex polymerization^25^.

Here we present a suite of rule-based models of clathrin polymerisation with increasing complexity, starting from a very basic model where the molecule has three equally reactive binding sites to a more advanced model reproducing realistic triskelia clathrin structure. We examined the correspondence of each model’s behavior with the existing theoretical models while sampling from a wide range of parameter values.

We found that although the basic model exactly reproduces Flory’s findings, it is unable to provide the amounts of 5- and 6- member rings required for cage formation and, therefore, it fails to reproduce clathrin vesicle formation. A revised model with a more realistic clathrin structure that explicitly supports predominant closure of pentagons and hexagons allows 3D cage formation and permits the evolution of flat 2D clathrin patches into a 3D cage structures by shifting the ratio of the pentagon/hexagon dissociation constants.

## Methods

### Models and simulation

We used the Kappa language^30^, a member of the family of rule-based modeling languages, for building the models. All models were simulated by KaSim3.5 (http://dev.executableknowledge.org/). We used Kappa extensions where appropriate, e.g the MetaKappa (https://github.com/kappamodeler/metakappa) extension for building the first model to handle the combinatorial explosion caused by three equal binding sites (see Appendix for details). Also, we use the RKappa extension^31^ for sampling the large parameter space, statistical analysis of simulation results, global sensitivity analysis (GSA) and visualization of the Kappa molecular structures as more comprehensible 2D and 3D graphs.

We first investigated the capability of rule-based models to reproduce clathrin cage structures based on random self-assembly processes. For this we assume that clathrin triskelia interact in 3D, in a well-mixed solution and all binding sites of the clathrin triskelia are assumed to be identical. Due to the combinatorial nature of the clathrin molecule association, the size of aggregates is unbounded and limited only by the amount of available substrate.

We started with a reduced model of triskelia monomers similar to Perelson and Goldstein’s equilibrium and continuous model^21^, in which monomers carry three identical equally reactive binding sites. Two variants of this model were implemented in the rule-based Kappa language to investigate the polymerization of branched complexes from a single class of trivalent agents under ‘rings allowed’ and ‘rings forbidden’ conditions similar to that proposed by^20^ (Model 1).

We then developed a more elaborate model, based on clathrin monomers, that considers triskelia as a predefined complex of three monomers. This model more accurately reproduces the structure of clathrin with distinct legs and binding sites along with specified defined steric and chirality constraints (Model 2). It also contains explicit rules describing formation of penta- and hexagonal rings and demonstrates the dynamics of closed cage structure formation. All the models presented here are kinetic and do not include notions of space. However these could be added by use of existing extensions like SpatialKappa^26^ or Geometric Kappa^32^ if required later.

### Equireactive trivalent agent model

**Figure 1:**
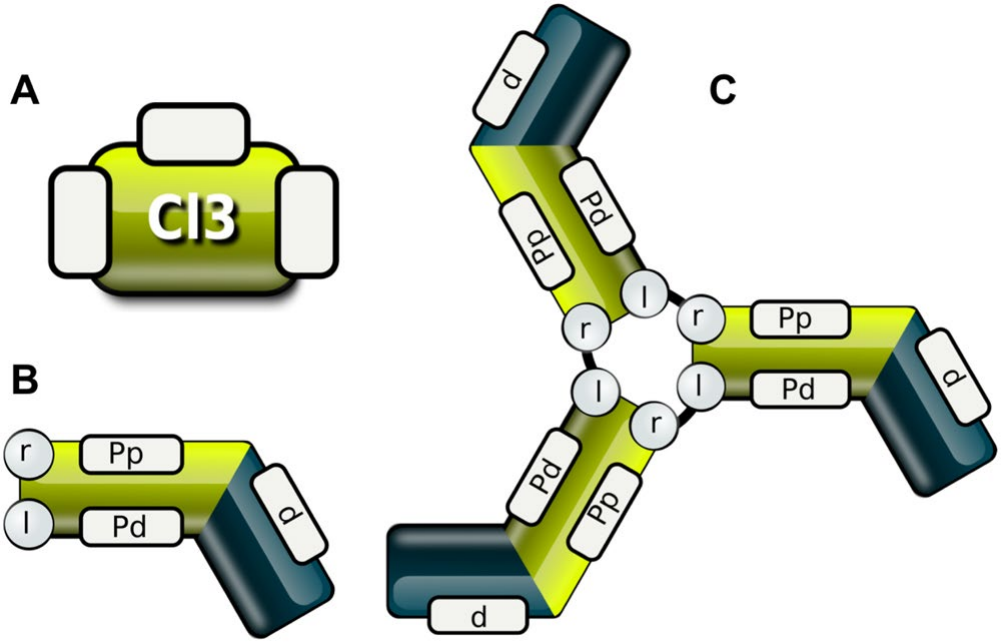
Structure of agents for Model 1 and Model 2. Three identical binding sites in a simple agent (**A**) interact with each other to form a lattice (Model1). Monomer (**B**) of detailed Model 2 has two sites to form the triskelia hub (l,r) and three sites to interact with other triskelia (d, Pd, Pp) (**C**).

In the first model (Model 1) we simplify the realistic triskelia structure of clathrin to the trivalent agent Cl3 with three identical binding sites. This is effectively a kinetic version of the model described by Perelson and Goldstein in 1985^16,21^ (Fig. 1A, Supplementary Data). As clathrin is known to aggregate on the membrane, we assume that with complex growth its ability to diffuse would decrease. Thus, in our configuration complex growth happens preferentially via addition of new monomers rather than merging of existing complexes, in the same way as in Perelson and Goldstein.

The (kappa) rule looks as follows:

$$ \mbox{`} proximalbinding\mbox{'}a(A,A,A),a(A)- > a(A!1,A,A),a(A!1)@ \mbox{`} pbk\mbox{'}(0),$$

where ‘*pbk’* is the rate of binding.

To ensure stability of the rings in clathrin complexes we make an assumption that molecules with three occupied binding sites cannot dissociate. Thus, dissociation is only possible at the periphery of the complex when at least one binding site is/remains free.

$$ \mbox{`} proximaldissociation\mbox{'}a(A!1,A),a(A!1)- > a(A,A),a(A)@ \mbox{`} pdk\mbox{'},$$

where *‘pdk’* is the rate of dissociation.

This rule partially contradicts the work of Perelson and Goldstein, where the dissociation is possible only at the monomer level. However, the rule includes the dissociation of terminal monomers as a special case.

We studied the random polymerisation of trivalent monomers under two traditional Flory- Stockmayer assumptions: ‘ring forbidden’ (Model 1 A) and ‘rings allowed (Model 1B).

In the case of Model 1 A (‘rings forbidden’), the intramolecular bonds between the binding sites of the same polymers are not allowed as the only free agent (with all three sites non-occupied) can bind the polymer. The detailed models for the original Perelson’s model and its two Kappa implementations: Model 1 A and Model 1B are presented in Supplementary Data.

In the case of Model 1B (‘ring allowed’) intra-molecular reactions are allowed, so that rings of different sizes may occur. As in^20,21,33^ reactions occur with an equal probability for each of the free binding site to react until the reaction extent R_ext_ = 1, which means that all binding sites are fully occupied. Although cubical structures of clathrin were observed experimentally under special conditions^34^, the formation of rings of size 4 and less is not reported under conditions approximating intracellular environments. Hence we set a specific constraint on the polymer chain ability to make intramolecular bonds only when ring size (nring) exceeds 5 bonds in length.

$$ \mbox{`} ringclosure\mbox{'}a(A),a(A)- > a(A!1),a(A!1)@ \mbox{`} pring\mbox{'}(0.0: \mbox{`} nring\mbox{'})$$

In the rule above *‘pring’* is a rate of ring closure, while *‘nring’* refers to the minimal number of bonds in the ring (set to 5 in this case). The constraints enforce limitations on the condition of equal reactivity to be always fulfilled; yet the probability to close a short ring within a large complex is quite small. We also assume the equilibrium constants for initiation, elongation and branching are equal.

### Triskelia model

To generate a more realistic model we next considered clathrin monomers and their structural properties. Each monomer consists of a proximal region (“P”, light green in Fig. 1B), which contains a binding domain on its “right”, long part (“r”) and “left”, short part (“l”), and the distal region (“d”, dark green in Fig. 1B). Domains in the proximal region facilitate the internal binding of monomers to form trimers. The additional binding sites “Pp” and “Pd” in the proximal region allow binding amongst different triskelia. Binding rules presume the ‘right’ part of one monomer can only bind to the “left” part of another, and so forth to make correct triskelia structures (Fig. 1C).

In kappa language this is expressed in the following way:

$$Cl(l!1,r!2),Cl(r!1,l!3),Cl(r!3,l!2)$$

“Cl” refers to a single clathrin molecule with proximal right (“r”) and left (“l”) binding site. All distal parts of the long legs are oriented in one direction, showing a clockwise drift/turn (Fig. 1C).

Once assembled, triskelia form the structural unit for the polymerisation process, which is governed by the interaction of domains localised on the right, long leg of each monomer. These are: a proximal (Pp), a distal “receiving” (Pd) and distal “giving” (d) domain. Based on the given clathrin triskelia structure, formation of one bond utilizes four triskelia simultaneously: two monomers bind with their proximal parts, and two form additional bonds with their distal parts (see Supplementary Data for triskelia binding code and a visualization). As was shown by den Otter *et al*.^17^ and Fotin *et al*.^35^, the proper orientation of all four legs is vital for formation of closed structures. Initial polymerization steps along with the model rules are presented in detail in Supplementary Data.

In addition to the binding rule, a few specific rules enforce the closure/formation of pentagons and hexagons. Dissociation is implemented as follows. Closed rings cannot be reopened. At least one monomer needs to be unbound for dissociation to happen. Details can be seen in the model code in the Supplementary Data, which shows the rules used in the current model version.

### Data Availability

All data generated or analysed during this study are included in this published article (and its Supplementary Information files).

## Results

We investigated the ability of rule-based models to reproduce the clathrin cage structures based on a random self-assembly process. Specifically, two traditional Flory- Stockmayer conditions: “rings forbidden” and “rings allowed” were applied separately, similar to^20^. All models were simulated 5000 times with parameter ranges shown in Table 1.

**Table 1:** Ranges for parameter space exploration.

Structure	Max number
g531	2
g532	4
g541	16
g551	250
g643	2
g651	4
g661	36

### Trivalent model

In the first model we used a simplified triskelia structure of clathrin with a trivalent agent Cl3 containing three identical binding sites with equal reactivity, similar to the Perelson and Goldstain model in 1985^21^.

The key parameters that have been analyzed are (see also^21^):

1 $${{R}}_{{e}xt}=\frac{2\ast {{N}}_{{bond}}}{3\ast {amount}}$$

2 $$\alpha =6\ast K\ast {C}_{\tau }=6\ast \frac{pbk\ast ({N}_{A}\ast V)}{pdk}=6\ast {N}_{\tau }\ast \frac{pbk}{pdk}$$

where *R*_*ext*_ - reaction extent, ***α*** - nondimensional equilubrium constant, *N*_*bond*_ - the number of bonds in the polymer, and *K* – the equilibrium constant. *C*_*t*_ and *N*_*t*_ describe the total concentration and total number of monomers (respectively), *amount* – amount of available triskelia.

**Figure 2:**
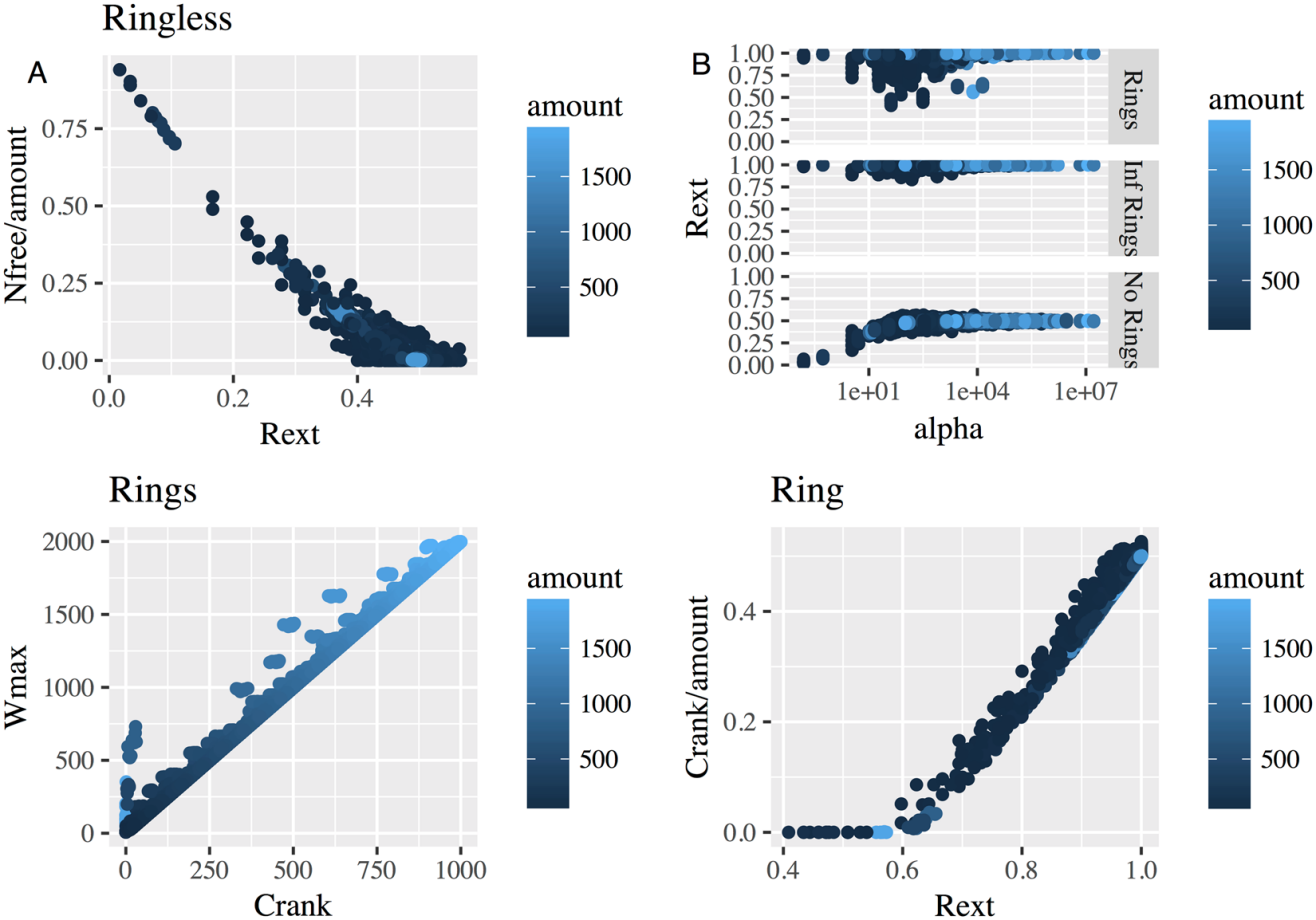
Simulation (5000 parameter sets) of the trivalent model with “ring forbidden” (**A**) and “ring allowed”. (**C**,**D**) assumptions. (**A**) The number of free agents Nfree decreases with Rext and trends to 0 at Rext = 0.5. (**B**) The relationship of alfa and Rext under different experimental conditions: “no ring”, “ring” and “infinite ring”. (**С**) The dependency between the size of the largest aggregate and cyclomatic number under “ring allowed” condition. (**D**) Relationship between reaction extent and loop structure under “rings allowed”.

We showed that in the “ring forbidden” setup, the distribution of free clathrin with dependence on *R*_*ext*_ exactly followed the prediction of Perelson’s theory (Fig. 2A and B). The vast majority of parameter sets in “ring forbidden” are grouped around *R*_*ext*_ = 0.5, and the dependency between *R*_*ext*_ and *N*_*bond*_*/N*_*t*_ is linear. We found that *R*_*ext*_ never exceeded the theoretical limit of gel formation (Fig. 2B) while in most of the “ring allowed” instances, reactions stopped only when the available binding sites were saturated (Fig. 2B and D).

To explore the types of complexes our simulations produced, we calculated the size of the largest aggregate (***W***_max_)and the number of the rings in the system. The latter was estimated as the cyclomatic number of the clathrin graph, which is the number of bonds that need to be removed to form an acyclic graph:

3 $${C}_{rank}={E}_{g}-{V}_{g}+{C}_{g}$$

with *E*_*g*_ number of edges and *V*_*g*_ number of nodes in the graph. *C*_*g*_ is the number of connected components in the graph. We found that the number of rings in the system ***C***_***rank***_ almost always reached the theoretical limit (Fig. 2D), where the total number of monomers was equal to the size of the largest aggregate (***W***_***max***_) in agreement with analysis from Falk *et al*.^20^.

In agreement with^20^, when intramolecular bonds are allowed (Model 1b) ring formation only starts after gel structure formation (Fig. 2D), when the reaction extent reaches the 0.5 threshold. This means that in the simple agent model closed cages would be formed only when 7/8 of the available clathrins form a large single complex.

**Figure 3:**
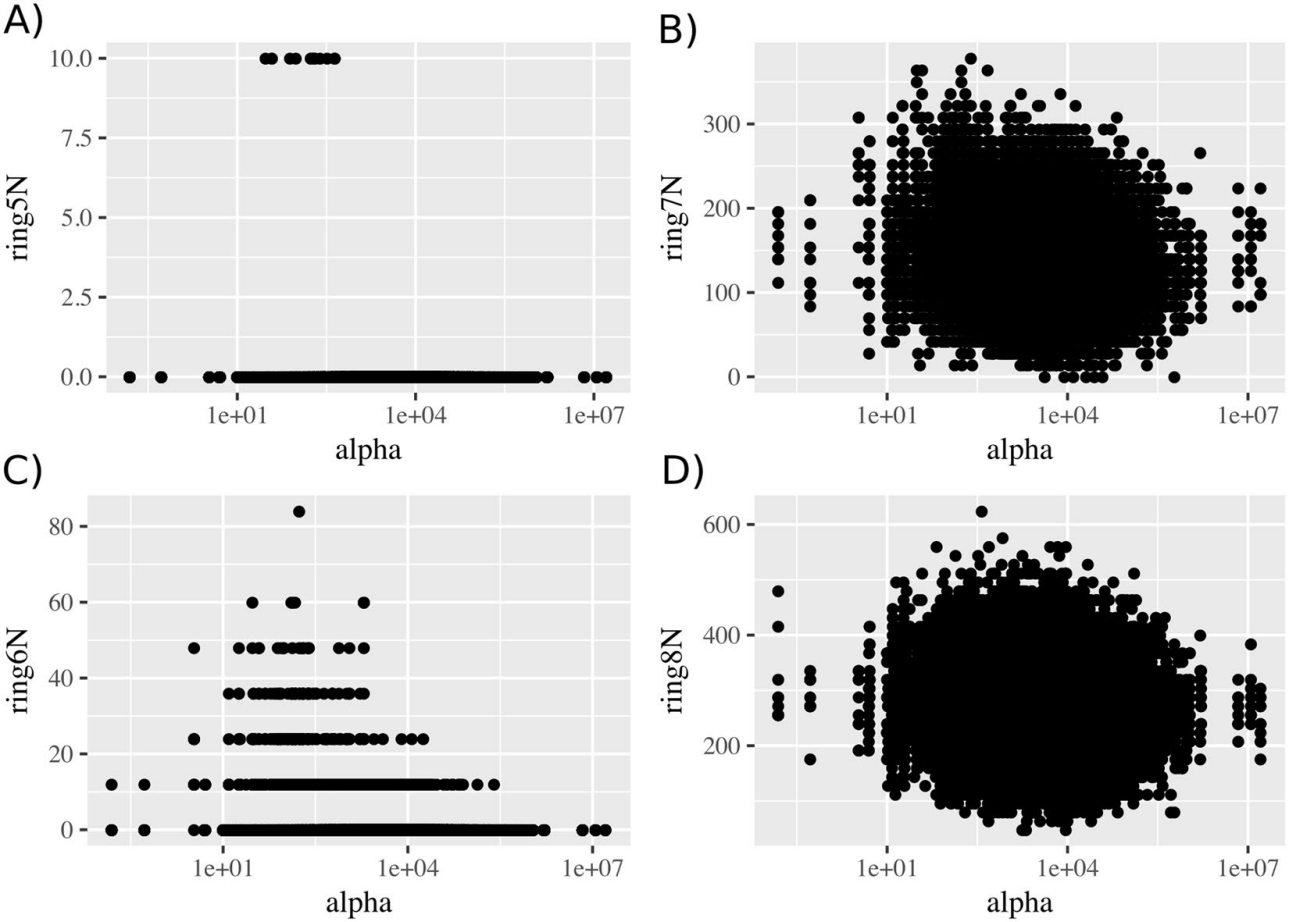
Distribution of different cyclic structures obtained from 5000 simulations. (**A**) 5-membered rings. (**B**) 6-membered rings. (**C**) 7-membered rings. (**D**) 8-membered rings.

Further analysis (Supplementary Data) shows that probability of the ring closure grows with the size of the ring. Therefore, the number of short rings (pentagons and hexagons) is quite low even when we set the rate of the ring closure reactions to infinity (Supplementary Data and Fig. 3). Therefore we conclude that the simple model is not able to describe the closed cage structures, as the clathrin geometry provides the optimal mutual disposition of the monomers only when 5- and 6-membered rings are formed. To resolve this we developed a more plausible model as follows.

### Triskelia model

Model 2 described above corresponds to a more realistic structure of clathrin with distinct regions within the monomer and respective binding sites that reflect the experimental literature^10,12^. We also introduced a specific rule for orientation of the monomers to ensure that the “right” site of one monomer binds the “left” side of another. This preserves the correct geometry of triskelia and chirality of the monomers. To ensure we obtain realistic clathrin complexes, 5- and 6- ring closure reactions were explicitly specified.

We started with parameter sampling for the model. To ensure comparability between simulations we used the same parameter sets as before by assigning the ring closure rate the value of “pring” to both hexagons and pentagons. Again, the two cases - “ring forbidden” and “ring allowed” were investigated.

**Figure 4:**
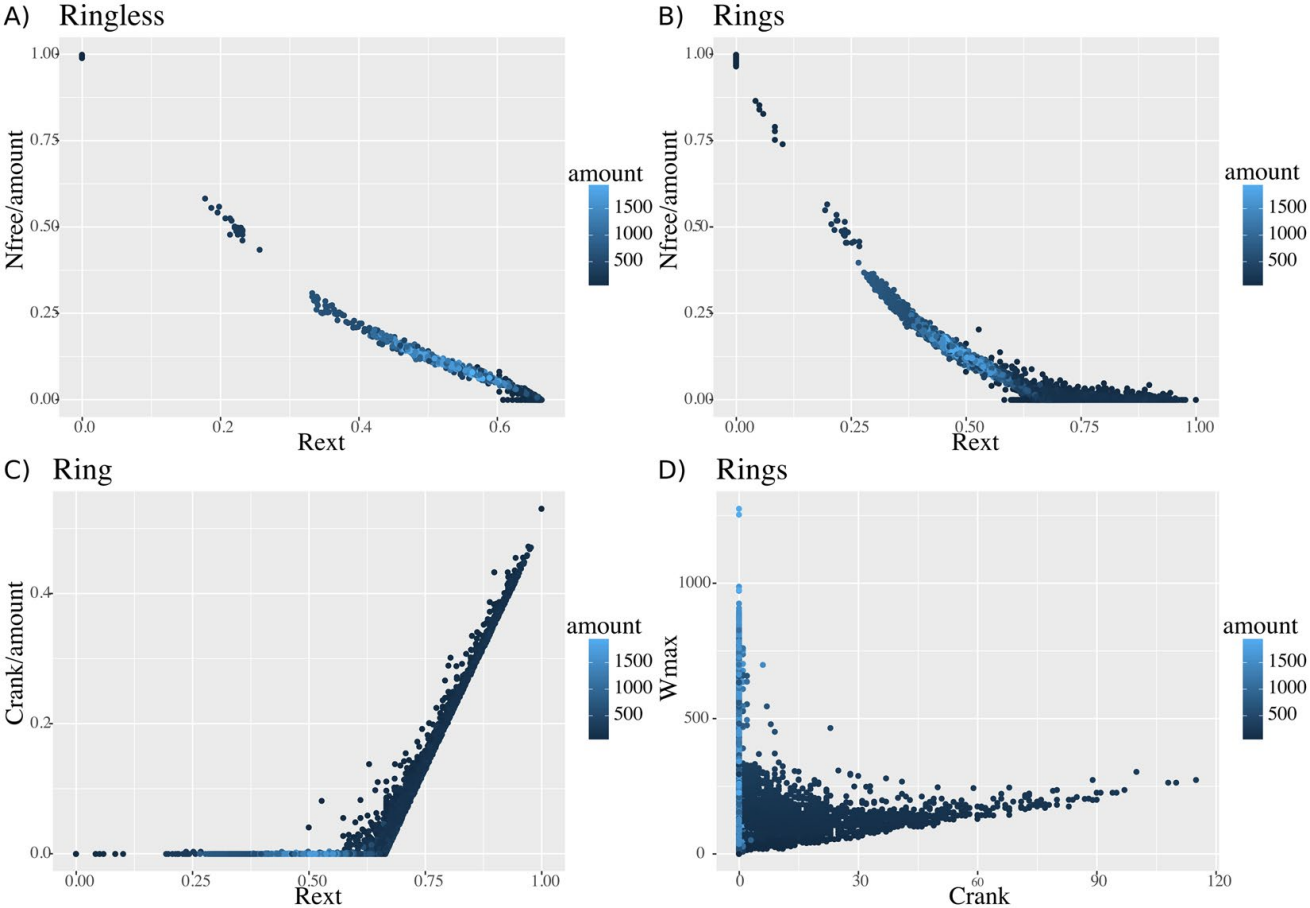
Results of simulation of “ring forbidden” and “ring allowed” models. (**A**) The number of free agents Nfree decreases with Rext towards 0 at Rext = 0.6 for “rind forbidden” model. (**B**) The number of rings (cyclomatic number of the graph) per triskelia in the “ring allowed” model.

The behavior of the “ring forbidden” version of Model 2 is clearly similar to the behavior of the Model 1 and theoretical predictions of Perelson (Fig. 4A). The number of free triskelia monotonically decreases towards zero at *R*_*ext*_ = 0.6. The difference between the theoretical prediction of 0.5 and the observed value is explained by the association rule in the Model 2, which does not prevent associations of clusters and therefore does not follow the monomer attachment mechanisms considered in Perelson^21^. Association between clusters results in a higher numbers of triskelia with all their legs involved in the complex formation, which in turn prevents their dissociation.

The “ring allowed” version of the Model 2 (Fig. 4B) follows the same scenario as Model 1 (Fig. 2D) and behaves as predicted by theory^20^. Ring formation starts only after solution to gel transition at *R*_*ext*_ = 0.6. Contrary to the Model 1, the number of rings does not grow linearly with the size of the complex (Fig. 2C). Instead, due to the system not being allowed to form rings of arbitrary size, we obtain many small complexes with few or no rings (Supplementary Figure 2).

**Figure 5:**
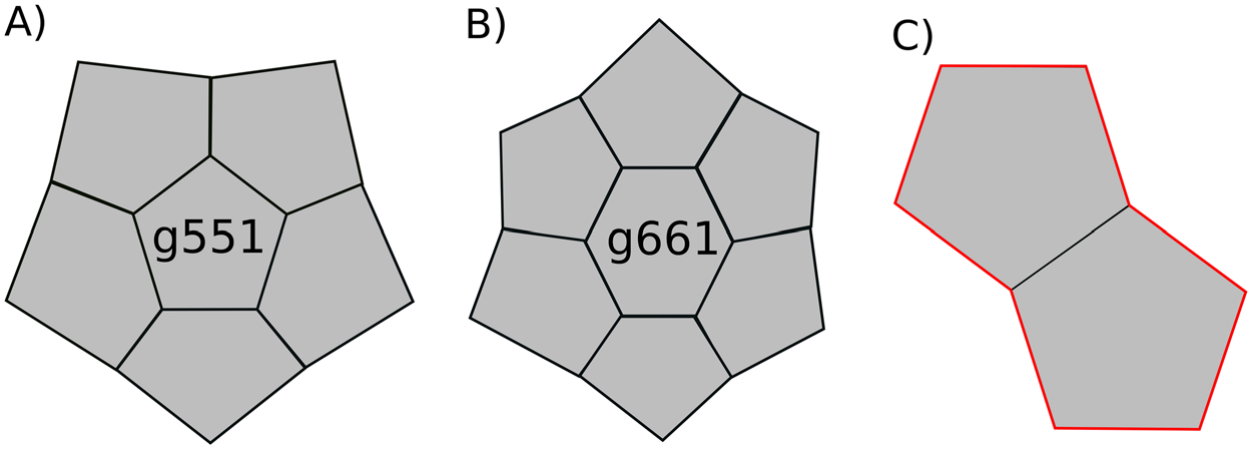
Most populated structures obtained in 5000 simulations of the unconstrained model. (**A**) Most populated pentagon structure. (**B**) Most populated hexagon structure. (**C**) An 8-ring formed by two pentagons.

For simplicity of simulation and comparison with Model 1 we did not introduce separate kinetic constants for 5- and 6-ring closure. As a result, the vast majority of the rings in our simulations are pentagons. Nevertheless we observed a number of hexagons as well. The relatively high number of octagons observed is a consequence of high number of 5-rings, as hull of two adjacent pentagons can form an octagon (Fig. 5С).

To explore the geometry of complexes, which contain 5- and 6-rings we used a set of all possible combinations of pentagons and hexagons as described in^22^. Table 2 shows that pentagons tend to form adjacent dodecahedron-like structures (see g551, Fig. 5A, Supplementary Figure 5), while hexagons are most often surrounded by pentagons as visualized in structure g661 (Fig. 5B). We found no clear distinction between ring forming and ring preventing values in parameter sets (Supplementary Figure 3). To further investigate which parameters influence the ring formation the most we performed GSA on Model 2 with the “ring allowed” condition (Supplementary Table 1). We thus concluded that Model 2 is able to produce various structures of different shapes (Fig. 5) without the initial constraints, but that they do not all necessarily end up being cage-like structures.

**Table 2:** Number of pent-Rings (g5) and hex-Rings (g6) found in GSA of the “ring allowed” version of the Model 2.

Parameter description	Parameter name	min	max
Association rate constant	*pbk*	10 E-6	1.00
Dissociation rate constant	*pdk*	10 E-6	1.00
Ring closure rate constant	*pring*	10 E-6	1.00
Amount of available triskelia	*amount*	10 E2	10 E4

**Figure 6:**
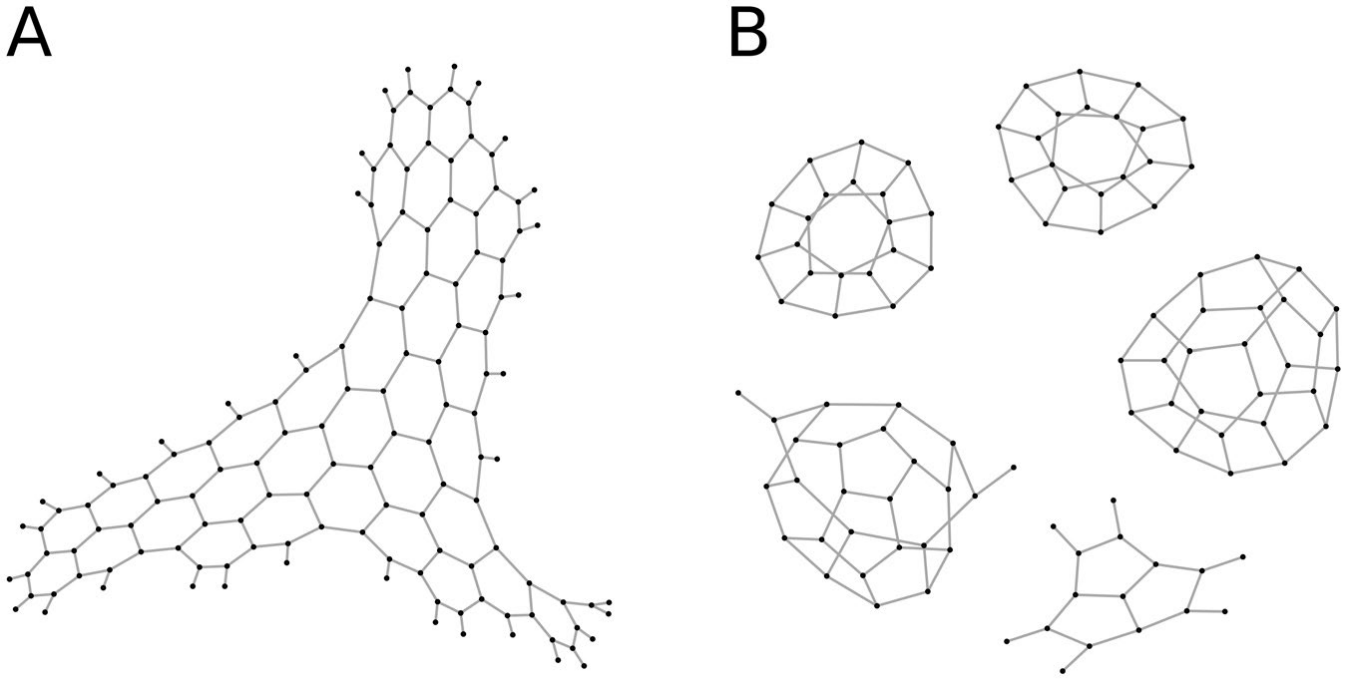
Results of model simulation with different Kd for 5 and 6-membered rings. (**A**) Only hexagons are allowed. (**B**) Rate closure for 5-rings was set to infinite.

The type of clathrin cage formed *in vivo* is known to depend on the ratio of pentagons and hexagons^3,22^. Moreover, planar clathrin consists of just hexagons. As an example we tested to see whether our model could be reconciled wit the invagination mechanism (e.g. described in Avinoam *et al*.^5^). Avinoam’s (2015) mechanism requires the presence of pentagons. To reproduce this we tuned the rate constants for pentagon and hexagon closure and changed the equilibrium of association and dissociation rates for them. First we simulated the model where only 6-rings were allowed by setting the 5-ring closure reaction to 0 to form a planar structure (Fig. 6A and Supplementary movie 1). When the reaction extent was close to 1, 5-ring closure was allowed by adjusting rate constant to non-zero value. With a rate of closure for 5- and 6-ring close to each other we observed invaginations, but they never reached the scissing stage so that the completely closed structure never occurred (Supplementary movie 2). At this point we set the rate of closure for 5-rings to infinite and after 10^4^ events we obtained the structures shown in Fig. 6B and Supplementary Movie 3.

To evaluate the influence of rates of pentagon and hexagon closure/disruption we performed GSA on the model starting with a flat hexagonal mesh (Supplementary Table 2). Here, b and d are the coefficients defining the extent to which pentagon closure is faster than hexagon closure (b), and hexagon compared to pentagon dissociation (d); rng5 and rng6 are the ratios of ring closure to ring disruption for pentagons and hexagons, respectively. For each parameter the significance level is calculated as described in^36^. The rate of the pentagon closure did not significantly influence any property of the system, while the rate of hexagon dissociation appeared important for the size of the most frequent complex (*wNmax*) and the presence of hexagon-containing subgraphs (g501, g511, g521, g522, g601, g611, g621, g622, g631, g632, g633, g641, g642, g643 in Supplementary Tables 1 and 2)^22^. During the course of a simulation we were able to obtain different numbers of closed cages in almost half of the parameter sets, which indicates that the formation of flat structures requires additional constraints, while cage formation happens spontaneously^4^.

## Discussion

Computational models describing formation of clathrin-coated vesicles (CCVs)^2,17,23,37^ mostly focus on clathrin self-association or its association with the membrane. However, vesicle recycling is regulated via a large number of signalling processes^2,38^. Existing computational models struggle to incorporate these regulatory elements either because of high computational cost, which becomes prohibitive in case of incorporation of all involved protein types, or because the structure/type of the model can/does not include the reactions controlled by regulatory systems. For example, the equilibrium model^21^ considered growth of pits as a linear set of reactions, assuming that all three legs of the new triskelia in the pit assemble using the best possible free sites in the net. As shown by simulations in^17^ and confirmed in our Models 1 A and B this is not the case.

As was proposed in^39^, these signaling processes can be incorporated into models as a modification of clathrin association/dissociation rates. With these factors in mind we have developed a model capable of describing the formation of CCVs, avoiding the more resource expensive computational algorithms and using a modeling format familiar to the signal transduction modelling community.

Our first version of the model, which described clathrin as a trivalent agent demonstrated that formation of closed structures required an additional manual closure to achieve 5- and 6- rings. With the flexibility of the clathrin molecule and no evidence for energy differences between penta- and hexameric rings we saw no preferences towards either specific ring composition. Weak interactions, which have been proposed to have a major effect on the association of clathrin legs^39^, and comparatively low bending energy of the clathrin lattice suggest that when on the flat part of the membrane, clathrin will create a flat hexagonal lattice. That process was considered in^3^, where clathrin was modeled as hexagonal lattice with 5- and 7-sided rings occurring as defects, but the study only considered the equilibrium state, whereas in our analysis we were able to investigate the kinetics of the process. Although the “canonic” mechanism of clathrin pits formation proposes constant curvature growth as a function of clathrin polymerization^40^, the evolution of curved clathrin structures from flat plaque has also some supporting experimental evidence^5,38^. The recent study of Leyton-Puig *et al*.^7^ reports the ability of clathrin plaques to act as hubs for CME and proposes actin polymerisation and actin-based adhesion are major regulating factors for their remodeling^7^.

Our model shows that switching pentagon ring formation on/off allows the process to switch between planar patches and closed cages. *In vivo*, this switching could be driven by changes in physical properties of the membrane or by additional regulatory mechanisms^1,37,41^.

In our model we assume the size and the shape of the clathrin lattice to be controlled by three processes: i) the association/dissociation of triskelia; ii) the 5-ring formation/dissociation and iii) the 6-ring formation/dissociation. Several other factors are known to influence the cage and coat formation and dissociation^42,43^. For example in^44^, the main difference in pentagon and hexagon closure is attributed to the stiffness of the underlying membrane, while in^41^ the rigidity variation of the clathrin net itself is explained by binding to an adaptor protein (AP2, AP3, AP180)^8,45^. Their influence on clathrin coat formation has been studied in distinct experimental setups and binding to clathrin has been confirmed. Due to their influence on clathrin triskelia structure and hence their ability to influence coat formation it might be debatable if their main role is in maintaining a flat structure or “forcing”/inducing the formation of vesicles. This mechanism could be easily embedded into the model (see the example in Supplementary Data).

The clathrin light chain is an additional part of the triskelia, which connects to the heavy chain in the region extending from the self-association domain to the knee^39^. One of the possible conformations can force the knee to bend in a direction that inhibits cage formation. This inhibitory effect is thought to be regulated (inhibited) by interaction with Ca ions or by lowering the pH^39^. The light chain also influences the rigidity of the clathrin lattice and its ability to bend the lipid membrane at low temperature^4^. The light chain contains 19 serines that are potential kinase targets (GRK2) and phosphorylation of the light chain has been proposed as a discriminator for different cargo inclusion in the vesicle^46^. An example of how the model can be extended to incorporate the above mechanism is presented in Supplementary Data.

The rule-based approach we have used allows us to build and compare kinetic models that describe different possible mechanisms of clathrin cage formation, from direct assembly from monomers at the vesicle budding site to the invagination of flat membrane plaque. More in depth functional details such as the role of N-WASP through Arp2/3^7^ can help to expand models and gain deeper insights. Hence, our implementation is easily extendable allowing the future inclusion of more detailed mechanistic models of CME regulation.

## Electronic supplementary material

Supplementary information

Supplementary movie 1

Supplementary movie 2

Supplementary movie 3

## References

[1] McMahon HT, Boucrot E (2011). Molecular mechanism and physiological functions of clathrin-mediated endocytosis. Nat. Rev. Mol. Cell Biol..

[2] Jung N, Haucke V (2007). Clathrin-mediated endocytosis at synapses. Traffic.

[3] Jin AJ, Nossal R (1993). Topological mechanisms involved in the formation of clathrin-coated vesicles. Biophys. J..

[4] Dannhauser PN (2015). Effect of clathrin light chains on the stiffness of clathrin lattices and membrane budding. Traffic.

[5] Avinoam O, Schorb M, Beese CJ, Briggs JAG, Kaksonen M (2015). Endocytic sites mature by continuous bending and remodeling of the clathrin coat. Science.

[6] Dannhauser PN, Ungewickell EJ (2012). Reconstitution of clathrin-coated bud and vesicle formation with minimal components. Nat. Cell Biol..

[7] Leyton-Puig D (2017). Flat clathrin lattices are dynamic actin-controlled hubs for clathrin-mediated endocytosis and signalling of specific receptors. Nat. Commun..

[8] Smith SM, Baker M, Halebian M, Smith CJ (2017). Weak Molecular Interactions in Clathrin-Mediated Endocytosis. Front Mol Biosci.

[9] Saheki Y, De Camilli P (2012). Synaptic vesicle endocytosis. Cold Spring Harb. Perspect. Biol..

[10] Fotin A (2004). Structure of an auxilin-bound clathrin coat and its implications for the mechanism of uncoating. Nature.

[11] Girard M, Allaire PD, McPherson PS, Blondeau F (2005). Non-stoichiometric relationship between clathrin heavy and light chains revealed by quantitative comparative proteomics of clathrin-coated vesicles from brain and liver. Mol. Cell. Proteomics.

[12] Kirchhausen T, Owen D, Harrison SC (2014). Molecular structure, function, and dynamics of clathrin-mediated membrane traffic. Cold Spring Harb. Perspect. Biol..

[13] Fotin A (2006). Structure determination of clathrin coats to subnanometer resolution by single particle cryo-electron microscopy. J. Struct. Biol..

[14] Pearse BM (1975). Coated vesicles from pig brain: purification and biochemical characterization. J. Mol. Biol..

[15] Pastan I, Willingham MC (1985). The pathway of endocytosis. J. Mol. Biol..

[16] Pearse BM, Crowther RA (1987). Structure and assembly of coated vesicles. Annu. Rev. Biophys. Biophys. Chem..

[17] den Otter WK, Renes MR, Briels WJ (2010). Asymmetry as the key to clathrin cage assembly. Biophys. J..

[18] den Otter WK, Briels WJ (2011). The generation of curved clathrin coats from flat plaques. Traffic.

[19] Matthews R, Likos CN (2013). Structures and pathways for clathrin self-assembly in the bulk and on membranes. Soft Matter.

[20] Falk M, Thomas RE (1974). Molecular size distribution in random polyfunctional condensation with or without ring formation: computer simulation. Can. J. Chem..

[21] Perelson AS, Goldstein B (1985). The equilibrium aggregate size distribution of self-associating trivalent molecules. Macromolecules.

[22] Schein S, Sands-Kidner M (2008). A geometric principle may guide self-assembly of fullerene cages from clathrin triskelia and from carbon atoms. Biophys. J..

[23] Banerjee A, Berezhkovskii A, Nossal R (2012). Stochastic model of clathrin-coated pit assembly. Biophys. J..

[24] Danos, V., Feret, J., Fontana, W., Harmer, R. & Krivine, J. Rule-based modelling and model perturbation. *Transactions on Computational Systems Biology XI* 116–137 (2009).

[25] Monine MI, Posner RG, Savage PB, Faeder JR, Hlavacek WS (2010). Modeling multivalent ligand-receptor interactions with steric constraints on configurations of cell-surface receptor aggregates. Biophys. J..

[26] Sorokina O, Sorokin A, Armstrong JD, Danos V (2013). A simulator for spatially extended kappa models. Bioinformatics.

[27] Danos, V., Feret, J., Fontana, W. & Krivine, J. Scalable simulation of cellular signaling networks. *Computational Methods In Systems Biology*, *Proceedings* 139–157 (2009).

[28] Colvinr J (2010). RuleMonkey: software for stochastic simulation of rule- based models. BMC Bioinformatics.

[29] Sneddon MW, Faeder JR, Emonet T (2011). Efficient modeling, simulation and coarse-graining of biological complexity with NFsim. Nat. Methods.

[30] Danos, V., Feret, J., Fontana, W. & Krivine, J. Abstract interpretation of cellular signalling networks. *Verification*, *Model Checking*, *and Abstract Interpretation* 83–97 (2008).

[31] Sorokin, A., Sorokina, O. & Armstrong, J. D. RKappa: Statistical sampling suite for Kappa models. in Hybrid Systems Biology (eds. Maler, O., Halasz, A. & Piazza, C.) 128–142 (Springer, 2015).

[32] Danos V, Honorato-Zimmer R, Jaramillo-Riveri S, Stucki S (2015). Rigid Geometric Constraints for Kappa Models. Electron. Notes Theor. Comput. Sci..

[33] Goldstein B, Perelson AS (1984). Equilibrium theory for the clustering of bivalent cell surface receptors by trivalent ligands. Application to histamine release from basophils. Biophys. J..

[34] Sorger PK, Crowther RA, Finch JT, Pearse BM (1986). Clathrin cubes: an extreme variant of the normal cage. J. Cell Biol..

[35] Fotin A (2004). Molecular model for a complete clathrin lattice from electron cryomicroscopy. Nature.

[36] Marino S, Hogue IB, Ray CJ, Kirschner DE (2008). A methodology for performing global uncertainty and sensitivity analysis in systems biology. J. Theor. Biol..

[37] Muthukumar M, Nossal R (2013). Micellization model for the polymerization of clathrin baskets. J. Chem. Phys..

[38] Ungewickell EJ, Hinrichsen L (2007). Endocytosis: clathrin-mediated membrane budding. Curr. Opin. Cell Biol..

[39] Wilbur JD (2010). Conformation switching of clathrin light chain regulates clathrin lattice assembly. Dev. Cell.

[40] Lampe M, Vassilopoulos S, Merrifield C (2016). Clathrin coated pits, plaques and adhesion. J. Struct. Biol..

[41] Nossal R (2001). Energetics of clathrin basket assembly. Traffic.

[42] Böcking T, Aguet F, Harrison SC, Kirchhausen T (2011). Single-molecule analysis of a molecular disassemblase reveals the mechanism of Hsc70-driven clathrin uncoating. Nat. Struct. Mol. Biol..

[43] Doherty GJ, McMahon HT (2009). Mechanisms of endocytosis. Annu. Rev. Biochem..

[44] Shraiman BI (1997). On the role of assembly kinetics in determining the structure of clathrin cages. Biophys. J..

[45] Saleem M (2015). A balance between membrane elasticity and polymerization energy sets the shape of spherical clathrin coats. Nat. Commun..

[46] Ferreira F (2012). Endocytosis of G protein-coupled receptors is regulated by clathrin light chain phosphorylation. Curr. Biol..

