# Supplementary material for: Rule-based modelling provides an extendable framework for comparing candidate mechanisms underpinning clathrin polymerisation
Source: Sci Rep. 2018 Apr 4;8:5658. doi: 10.1038/s41598-018-23829-x (PMC5884807; doi:10.1038/s41598-018-23829-x)
Supplement: Supplementary file 1 — Supplementary information [file 41598_2018_23829_MOESM1_ESM.pdf]

SUPPLEMENT MATERIAL
Rule-based modelling provides an extendable framework for comparing candidate mechanisms underpinning clathrin polymerisation.

A Sorokin, K F Heil, J D Armstrong and O Sorokina

January 23, 2018

Perelson model

In the paper (1) Perelson and Goldstein consider statistical properties of simple model of three- valent agents aggregation. The definition of the model shown in 1-3:

$$ {}_0\mathrm{a} + \mathrm{a}_0 \xrightleftharpoons[\mathrm{k}_{0-}]{\mathrm{k}_{0+}} {}_1\mathrm{a}{-}\mathrm{a}_1 \tag{1} $$

$$ {}_1\mathrm{a}^{+}\ \mathrm{a}_0 \xrightleftharpoons[\mathrm{k}_{1-}]{\mathrm{k}_{1+}} {}_2\mathrm{a}{-}\mathrm{a}_1 \tag{2} $$

$$ {}_2\mathrm{a} + \mathrm{a}_0 \xrightleftharpoons[\mathrm{k}_{2-}]{\mathrm{k}_{2+}} {}_3\mathrm{a}{-}\mathrm{a}_1 \tag{3} $$

where ${}_0\mathrm{a}$ and a_0, ${}_1\mathrm{a}$ and a_1, ${}_2\mathrm{a}$ and a_2, ${}_3\mathrm{a}$ and a_3 represents an agent with zero, one and two binding sites occupied, respectively, dash between agents represents a bond formed. In this model growth or dissociation of the polymer chain caused by attachment or detachment of free monomer at the unoccupied site in the chain, so association of several chains into bigger one, or disruption of big chain into smaller parts is not possible. The key values in the Perelson model are dimensionless parameters

$$ c = \frac{C}{C_T} \tag{4} $$

$$ \alpha_0 = 6K_0C_T = 6\frac{k_{0+}}{k_{0-}}C_T \tag{5} $$

$$ \alpha = 6K_1C_T = 6\frac{k_{1+}}{k_{1-}}C_T \tag{6} $$

$$ \beta = \frac{3}{2}K_2C_T = \frac{3}{2}\frac{k_{2+}}{k_{2-}}C_T \tag{7} $$

where C is a concentration of free monomers and C_T – total concentration of monomers. We are restrict ourself with simplest case of this model when all sites are equivalent and all equilibrium constants are the same $K_0 = K_1 = K_2$. In this case, which called equireactivity, $\alpha_0 = \alpha = 4\beta$, and it could be shown that at equilibrium concentration of free monomers c and extent of reaction p has a value:

$$c = \frac{(1+2\beta)(1+8\beta)^{1/2} - (1+6\beta)}{16\beta^3} = \frac{2p(1-p)}{\alpha} \tag{8}$$

$$p = \frac{1+\alpha-(1+2\alpha)^{1/2}}{\alpha} \tag{9}$$

In that system there is gel/sol transition at which half of available binding sites are occupied so reaction extent become $p = 1/2$ and concentration of free monomers become $c = 1/8$. There is critical concentration $C_T^* = \frac{2}{3K}$, such that if $C_T < C_T^*$, gelation can never occur.

Model I

This model could be considered as slightly relaxed kinetic version of Perelson model implemented in Kappa. Triskelia has three equivalent binding sites and Kappa language do not allow identical sites, we have implemented the model with help of MetaKappa (2, 3):

Code 1: Metamodel I

```
1 %gen: a(A)
2 %conc: cl=a[A\{r g b}]
3
4 #binding of proximal domains
5 'proximal␣binding' a(A,A,A) , a(A) -> a(A!1,A,A) , a(A!1) @ 'pbk' (0)
6
7 #dissociation
8 'proximal␣dissociation' a(A!1,A) , a(A!1) -> a(A,A) , a(A) @ 'pdk'
```

First line creates a generic agent a with one binding site A, line 2 defines clathrin cl as an agent with three sites r, g, b each equivalent to the the site A of generic agent a. This model allow formation of new bond by free agent only (line 5 agent $a(A, A, A)$), similar to the Perelson model, but bond dissociation is allowed at any linear fragment (line 8 agent with one site free $a(A!1, A)$). There are two constants defined in the model: binding rate constant pbk and dissociation rate constant pdk.

For simulation and analysis model was split into three parts: (i) agents and observable definitions; (ii) reaction definition; and (iii) parameters definition.

Code 2: Model I, agent definition

```
%agent: cl(r,g,b)
```

```
2
3 %init: 'amount' cl(r, g, b)
4
5 %obs: 'FreeD' cl(r,g,b)
6 %obs: 'boundR' cl(r!_)
7 %obs: 'boundG' cl(g!_)
8 %obs: 'boundB' cl(b!_)
9
10 %mod: [E+] > 'amount' * 10 do $STOP "final_state"
11
12 %mod: repeat ([E+] [mod] 1000)=0 do $SNAPSHOT "
    clathrin_dot_cplx" until [false]
13
14 %def: "dumpIfDeadlocked" "yes"
```

We have added third parameter *amount* to analyse the role of agent availability. Line 12 force KaSim save structure of the reaction mixture every 1000 events into a separate files.

Metakappa convert the model Code 1 into reaction definition part of the full model by substitution of equivalent sites r, g, b instead of site A. After minimal modification, required to make model compliant with KaSim 3.4 syntax, reaction definition part of the Model II shown on the listing Code 3:

Code 3: Model I reaction definition

```
#'proximal binding' a(A,A,A) , a(A) -> a(A!1,A,A) , a(A
    !1) @ 'pbk' (0)
'pb.l:1.b,r,g/A.l:1.r/A' cl(b,r,g) , cl(r) -> cl(b!1,r,g)
     , cl(r!1) @ 'pbk' (0)
'pb.l:1.b,r,g/A.l:1.g/A' cl(b,r,g) , cl(g) -> cl(b!1,r,g)
     , cl(g!1) @ 'pbk' (0)
'pb.l:1.b,r,g/A.l:1.b/A' cl(b,r,g) , cl(b) -> cl(b!1,r,g)
     , cl(b!1) @ 'pbk' (0)
'pb.l:1.g,r,b/A.l:1.r/A' cl(g,r,b) , cl(r) -> cl(g!1,r,b)
     , cl(r!1) @ 'pbk' (0)
'pb.l:1.g,r,b/A.l:1.g/A' cl(g,r,b) , cl(g) -> cl(g!1,r,b)
     , cl(g!1) @ 'pbk' (0)
'pb.l:1.g,r,b/A.l:1.b/A' cl(g,r,b) , cl(b) -> cl(g!1,r,b)
     , cl(b!1) @ 'pbk' (0)
'pb.l:1.r,g,b/A.l:1.r/A' cl(r,g,b) , cl(r) -> cl(r!1,g,b)
     , cl(r!1) @ 'pbk' (0)
'pb.l:1.r,g,b/A.l:1.g/A' cl(r,g,b) , cl(g) -> cl(r!1,g,b)
     , cl(g!1) @ 'pbk' (0)
'pb.l:1.r,g,b/A.l:1.b/A' cl(r,g,b) , cl(b) -> cl(r!1,g,b)
     , cl(b!1) @ 'pbk' (0)

#'proximal dissociation' a(A!1,A) , a(A!1) -> a(A,A) , a(
```

```
    A) @ 'pdk'
'pd.l:1.r,b/A.l:1.r/A.b/A' cl(r!1,b) , cl(r!1) -> cl(r,b)
    , cl(r) @ 'pdk'
'pd.l:1.r,b/A.l:1.g/A.b/A' cl(r!1,b) , cl(g!1) -> cl(r,b)
    , cl(g) @ 'pdk'
'pd.l:1.r,b/A.l:1.b/A.b/A' cl(r!1,b) , cl(b!1) -> cl(r,b)
    , cl(b) @ 'pdk'
'pd.l:1.r,g/A.l:1.r/A.b/A' cl(r!1,g) , cl(r!1) -> cl(r,g)
    , cl(r) @ 'pdk'
'pd.l:1.r,g/A.l:1.g/A.b/A' cl(r!1,g) , cl(g!1) -> cl(r,g)
    , cl(g) @ 'pdk'
'pd.l:1.r,g/A.l:1.b/A.b/A' cl(r!1,g) , cl(b!1) -> cl(r,g)
    , cl(b) @ 'pdk'
'pd.l:1.g,b/A.l:1.r/A.b/A' cl(g!1,b) , cl(r!1) -> cl(g,b)
    , cl(r) @ 'pdk'
'pd.l:1.g,b/A.l:1.g/A.b/A' cl(g!1,b) , cl(g!1) -> cl(g,b)
    , cl(g) @ 'pdk'
'pd.l:1.g,b/A.l:1.b/A.b/A' cl(g!1,b) , cl(b!1) -> cl(g,b)
    , cl(b) @ 'pdk'
'pd.l:1.g,r/A.l:1.r/A.b/A' cl(g!1,r) , cl(r!1) -> cl(g,r)
    , cl(r) @ 'pdk'
'pd.l:1.g,r/A.l:1.g/A.b/A' cl(g!1,r) , cl(g!1) -> cl(g,r)
    , cl(g) @ 'pdk'
'pd.l:1.g,r/A.l:1.b/A.b/A' cl(g!1,r) , cl(b!1) -> cl(g,r)
    , cl(b) @ 'pdk'
'pd.l:1.b,g/A.l:1.r/A.b/A' cl(b!1,g) , cl(r!1) -> cl(b,g)
    , cl(r) @ 'pdk'
'pd.l:1.b,g/A.l:1.g/A.b/A' cl(b!1,g) , cl(g!1) -> cl(b,g)
    , cl(g) @ 'pdk'
'pd.l:1.b,g/A.l:1.b/A.b/A' cl(b!1,g) , cl(b!1) -> cl(b,g)
    , cl(b) @ 'pdk'
'pd.l:1.b,r/A.l:1.r/A.b/A' cl(b!1,r) , cl(r!1) -> cl(b,r)
    , cl(r) @ 'pdk'
'pd.l:1.b,r/A.l:1.g/A.b/A' cl(b!1,r) , cl(g!1) -> cl(b,r)
    , cl(g) @ 'pdk'
'pd.l:1.b,r/A.l:1.b/A.b/A' cl(b!1,r) , cl(b!1) -> cl(b,r)
    , cl(b) @ 'pdk'
```

Parameter definition part, which is trivial, is shown on the next listing:

Code 4: Model I parameter definition

```
%var: 'pbk' 0.519984665711463
%var: 'pdk' 0.25862499703294
%var: 'amount' 1867
```

Model I ring

The ring closure is the key for closed cage structure formation, so we have created model in which rings are allowed by additional reaction in the metamodel:

Code 5: Metamodel I ring

```
1 %gen: a(A)
2 %conc: cl=a[A\{r g b}]
3
4 #binding of proximal domains
5 'proximal_binding' a(A,A,A) , a(A) -> a(A!1,A,A) , a(A!1)
     @ 'pbk' (0)
6
7 #dissociation
8 'proximal_dissociation' a(A!1,A) , a(A!1) -> a(A,A) , a(A
    ) @ 'pdk'
9
10 #ring closure
11 'ring_closure' a(A) , a(A) -> a(A!1) , a(A!1) @ 'pring'
    (0.0:'nring')
```

Line 11 allows bond formation between any two agents with free site, when they are no closer than $nring$ bonds to each other. With that modification three parts of the model shown on the listings:

Code 6: Model I ring, agent definition

```
%agent: cl(r,g,b)

%var: 'nring' 5

%init: 'amount' cl(r, g, b)

%obs: 'FreeD' cl(r,g,b)
%obs: 'boundR' cl(r!_)
%obs: 'boundG' cl(g!_)
%obs: 'boundB' cl(b!_)

%mod: [E+] > 'amount' * 10 do $STOP "final_state"

%mod: repeat ([E+] [mod] 1000)=0 do $SNAPSHOT "
    clathrin_dot_cplx" until [false]

%def: "dumpIfDeadlocked" "yes"
```

Code 7: Model I ring reaction definition

```
#'proximal binding' a(A,A,A) , a(A) -> a(A!1,A,A) , a(A
    !1) @ 'pbk' (0)
```

```
'pb.l:1.b,r,g/A.l:1.r/A' cl(b,r,g) , cl(r) -> cl(b!1,r,g) , cl(r!1) @ 'pbk' (0)
'pb.l:1.b,r,g/A.l:1.g/A' cl(b,r,g) , cl(g) -> cl(b!1,r,g) , cl(g!1) @ 'pbk' (0)
'pb.l:1.b,r,g/A.l:1.b/A' cl(b,r,g) , cl(b) -> cl(b!1,r,g) , cl(b!1) @ 'pbk' (0)
'pb.l:1.g,r,b/A.l:1.r/A' cl(g,r,b) , cl(r) -> cl(g!1,r,b) , cl(r!1) @ 'pbk' (0)
'pb.l:1.g,r,b/A.l:1.g/A' cl(g,r,b) , cl(g) -> cl(g!1,r,b) , cl(g!1) @ 'pbk' (0)
'pb.l:1.g,r,b/A.l:1.b/A' cl(g,r,b) , cl(b) -> cl(g!1,r,b) , cl(b!1) @ 'pbk' (0)
'pb.l:1.r,g,b/A.l:1.r/A' cl(r,g,b) , cl(r) -> cl(r!1,g,b) , cl(r!1) @ 'pbk' (0)
'pb.l:1.r,g,b/A.l:1.g/A' cl(r,g,b) , cl(g) -> cl(r!1,g,b) , cl(g!1) @ 'pbk' (0)
'pb.l:1.r,g,b/A.l:1.b/A' cl(r,g,b) , cl(b) -> cl(r!1,g,b) , cl(b!1) @ 'pbk' (0)

#'proximal dissociation' a(A!1,A) , a(A!1) -> a(A,A) , a(A) @ 'pdk'
'pd.l:1.r,b/A.l:1.r/A.b/A' cl(r!1,b) , cl(r!1) -> cl(r,b) , cl(r) @ 'pdk'
'pd.l:1.r,b/A.l:1.g/A.b/A' cl(r!1,b) , cl(g!1) -> cl(r,b) , cl(g) @ 'pdk'
'pd.l:1.r,b/A.l:1.b/A.b/A' cl(r!1,b) , cl(b!1) -> cl(r,b) , cl(b) @ 'pdk'
'pd.l:1.r,g/A.l:1.r/A.b/A' cl(r!1,g) , cl(r!1) -> cl(r,g) , cl(r) @ 'pdk'
'pd.l:1.r,g/A.l:1.g/A.b/A' cl(r!1,g) , cl(g!1) -> cl(r,g) , cl(g) @ 'pdk'
'pd.l:1.r,g/A.l:1.b/A.b/A' cl(r!1,g) , cl(b!1) -> cl(r,g) , cl(b) @ 'pdk'
'pd.l:1.g,b/A.l:1.r/A.b/A' cl(g!1,b) , cl(r!1) -> cl(g,b) , cl(r) @ 'pdk'
'pd.l:1.g,b/A.l:1.g/A.b/A' cl(g!1,b) , cl(g!1) -> cl(g,b) , cl(g) @ 'pdk'
'pd.l:1.g,b/A.l:1.b/A.b/A' cl(g!1,b) , cl(b!1) -> cl(g,b) , cl(b) @ 'pdk'
'pd.l:1.g,r/A.l:1.r/A.b/A' cl(g!1,r) , cl(r!1) -> cl(g,r) , cl(r) @ 'pdk'
'pd.l:1.g,r/A.l:1.g/A.b/A' cl(g!1,r) , cl(g!1) -> cl(g,r) , cl(g) @ 'pdk'
'pd.l:1.g,r/A.l:1.b/A.b/A' cl(g!1,r) , cl(b!1) -> cl(g,r) , cl(b) @ 'pdk'
'pd.l:1.b,g/A.l:1.r/A.b/A' cl(b!1,g) , cl(r!1) -> cl(b,g)
```

```
    , cl(r) @ 'pdk'
'pd.l:1.b,g/A.l:1.g/A.b/A' cl(b!1,g) , cl(g!1) -> cl(b,g) , cl(g) @ 'pdk'
'pd.l:1.b,g/A.l:1.b/A.b/A' cl(b!1,g) , cl(b!1) -> cl(b,g) , cl(b) @ 'pdk'
'pd.l:1.b,r/A.l:1.r/A.b/A' cl(b!1,r) , cl(r!1) -> cl(b,r) , cl(r) @ 'pdk'
'pd.l:1.b,r/A.l:1.g/A.b/A' cl(b!1,r) , cl(g!1) -> cl(b,r) , cl(g) @ 'pdk'
'pd.l:1.b,r/A.l:1.b/A.b/A' cl(b!1,r) , cl(b!1) -> cl(b,r) , cl(b) @ 'pdk'

#'ring closure' a(A) , a(A) -> a(A!1) , a(A!1) @ 'pring' (0.0:'nring')
'rc.l:1.b/A.l:1.r/A' cl(b) , cl(r) -> cl(b!1) , cl(r!1) @ 'pring' (0.0:'nring')
'rc.l:1.b/A.l:1.g/A' cl(b) , cl(g) -> cl(b!1) , cl(g!1) @ 'pring' (0.0:'nring')
'rc.l:1.b/A.l:1.b/A' cl(b) , cl(b) -> cl(b!1) , cl(b!1) @ 'pring' (0.0:'nring')
'rc.l:1.g/A.l:1.r/A' cl(g) , cl(r) -> cl(g!1) , cl(r!1) @ 'pring' (0.0:'nring')
'rc.l:1.g/A.l:1.g/A' cl(g) , cl(g) -> cl(g!1) , cl(g!1) @ 'pring' (0.0:'nring')
'rc.l:1.g/A.l:1.b/A' cl(g) , cl(b) -> cl(g!1) , cl(b!1) @ 'pring' (0.0:'nring')
'rc.l:1.r/A.l:1.r/A' cl(r) , cl(r) -> cl(r!1) , cl(r!1) @ 'pring' (0.0:'nring')
'rc.l:1.r/A.l:1.g/A' cl(r) , cl(g) -> cl(r!1) , cl(g!1) @ 'pring' (0.0:'nring')
'rc.l:1.r/A.l:1.b/A' cl(r) , cl(b) -> cl(r!1) , cl(b!1) @ 'pring' (0.0:'nring')
```

Code 8: Model I ring parameter definition

```
%var: 'pbk' 0.519984665711463
%var: 'pdk' 0.25862499703294
%var: 'pring' 0.419548704526812
%var: 'amount' 1867
```

Model I infinite ring

To analyse the role of ring closure rate in the model behaviour variation of Model I ring was created with parameter $pring = \infty$. That means that once there is combination of free sites within the same complex separated by more than 5 bonds, the new bond between those sites will be created immediately. Together with Model I, Model I infinite

ring test extreme cases of ring closure.

Model II

This model was designed to address inability of simple models to represent formation of closed cage structures by clathrin triskelia. Geometry of the triskelia and successful Monte-Karlo simulations of the cage formation from rigid 3D particles (4) show that formation of penta- and hexameric rings should be part of the model. The number of possible combination of left hand sides of reactions makes this approach impractical for the model with triskelia as an agent. So we have decided to make an individual clathrin monomer an agent and consider triskelia as a complex. This approach also makes easy to deal with stereochemistry of triskelia: its geometry suppose two possible isomers, and in the (4) shown that system should consists of only one of them, otherwise formation of closed complexes is impossible.

In this model we encode known structure of clathrin monomer (5) and binding structure between different monomers during cage formation (4, 6). Special attention has been payed to the proper orientation of binding triskelia, which is of crucial importance as shown in (4).

In that model clathrin monomer has five sites, as shown on the Supplementary figure 1 (and line 8 on the listing Code 9): two to form hub by binding with monomer to the left l-site and to the righth r-site; one on the distal part of the leg – d-site to bind it to the proximal part of another clathrin; and two sites on proximal part of the leg: Pp to bind proximal part of another clathrin and Pd to bind distal part of another clathrin.

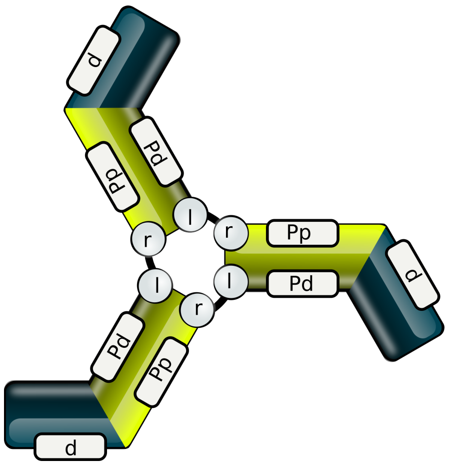

Supplementary Figure 1: Triskelion formed by three clathrin monomers as described in line 8 and 12 of listing Code 9.

Code 9: Model II, agent definition

```
1
```

```
# Clathrin monomer: r1 and r2 hub formation sites,
# d -- distal part of the leg,
# P -- proximal part of the  leg,
#      Pp -- binding site for another proximal leg
#      Pd -- binding site for distal leg

%agent: Cl(l,r,Pd,Pp,d)

%var: 'nring' 5

%init: 'amount' Cl(l!1,r!2),Cl(r!1,l!3),Cl(r!3,l!2)

%obs: 'FreeD' Cl(d)
%obs: 'FreePp' Cl(Pp)
%obs: 'FreePd' Cl(Pd)
%obs: 'bindLHS' Cl(r!1,Pd),Cl(l!1,Pp,d),Cl(l!2,Pp,d),Cl(r
    !2,Pd)
%obs: 'dissLHS' Cl(r!1,Pd!2),Cl(l!1,Pp!3,Pd,d!4),Cl(l!5
    ,Pp!3,d!2),Cl(r!5,Pd!4)

%mod: [E+] > 'amount' * 10 do $STOP "final_state"

%mod: repeat ([E+] [mod] 1000)=0 do $SNAPSHOT "
    clathrin_struct_cplx" until [false]

%def: "dumpIfDeadlocked" "yes"
```

The bond between triskelia are formed by association of two Pp sites of two clathrin monomers from different triskelia and Pd site with d site of the second monomer in triskelia as shown by red lines in the Supplementary figure 2. It could be seen in the figure that the orientation of the hub sites is important here to enforce asymmetry of binding, as described in a 'patchy' model in (4).

Code 10: Model II, association/dissociation reactions

```

 'binary␣binding' Cl(r!1,Pd),Cl(l!1,Pp,d),Cl(l!2,Pp,d),Cl
    (r!2,Pd) -> Cl(r!1,Pd),Cl(l!1,Pp!3,d),Cl(l!5,Pp!3,d)
    ,Cl(r!5,Pd) @ 'pbk' (0)

# binding of two proximal domains should cause formation
    of two p-d bonds as well
 'p-d␣closure' Cl(r!1,Pd),Cl(l!1,Pp!3),Cl(Pp!3,d) -> Cl(r
    !1,Pd!2),Cl(l!1,Pp!3),Cl(Pp!3,d!2) @ [inf]

 'proximal␣dissociation' Cl(r!1,Pd!2),Cl(l!1,Pp!3,Pd,d!4)
     ,Cl(l!5,Pp!3,d!2),Cl(r!5,Pd!4) -> Cl(r!1,Pd),Cl(l!1
```

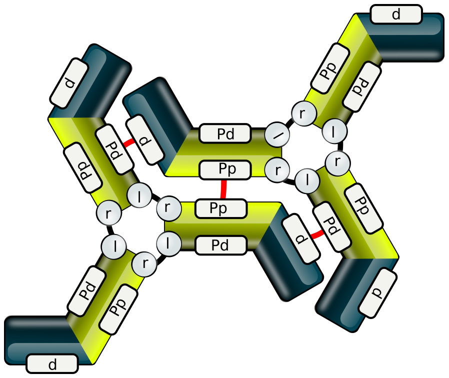

Supplementary Figure 2: Complex of two triskelia formed by interaction between three clathrin monomers. Red lines shows three bonds created by during binding of two triskelia as described in lines 2 and 5 of listing Code 10.

```
,Pp,Pd,d),Cl(l!2,Pp,d),Cl(r!2,Pd) @ 'pdk'
```

To simplify model definition only $Pp - Pp$ bond created during association reaction, corresponding two $Pd - d$ bond created separately with infinite rate constant. That does not influence simulation results, but allow more comprehensible rule definitions. To guarantee stability to the clathrin complexes we make an assumption that the molecule with three binding sites occupied cannot dissociate. Thus, dissociation is only possible when at least one leg of the triskelion has free Pp binding site.

The final and most important part of the Model II definition is the ring closure rules. Both reactions has the same rate constant $pring$ as shown on the listing Code 11:

Code 11: Model II, ring closure reactions

```
'hexagon␣closure' Cl(Pd!0,l!1,r!2),Cl(Pp!3,d!4,l!5,r!1)
    ,Cl(Pd!6,Pp!7,d!8,l!2,r!5),Cl(Pd!9,l!10,r!11),Cl(
    Pp,d,l!12,r!10),Cl(Pd,Pp!13,d!14,l!11,r!12),Cl(Pd!4,l
    !15,r!16),Cl(Pp!17,d!18,l!19,r!15),Cl(Pd!20,Pp!3,d!6
    ,l!16,r!19),Cl(Pd!8,Pp!27,d!28,l!29,r!30),Cl(Pd!31,l
    !32,r!29),Cl(Pp!7,d!0,l!30,r!32),Cl(Pp!13,d!9,l!33,r
    !34),Cl(Pd!14,Pp!17,d!20,l!35,r!33),Cl(Pd!18,Pp,d,l
    !34,r!35),Cl(Pp!27,d!31,l!36,r!37),Cl(Pd!28,Pp,d,l!38
    ,r!36),Cl(Pd,l!37,r!38)->Cl(Pd!0,l!1,r!2),Cl(Pp!3,d!4
    ,l!5,r!1),Cl(Pd!6,Pp!7,d!8,l!2,r!5),Cl(Pd!9,l!10,r
    !11),Cl(Pp!39,d!40,l!12,r!10),Cl(Pd!42,Pp!13,d!14,l
    !11,r!12),Cl(Pd!4,l!15,r!16),Cl(Pp!17,d!18,l!19,r!15)
    ,Cl(Pd!20,Pp!3,d!6,l!16,r!19),Cl(Pd!8,Pp!27,d!28,l!29
    ,r!30),Cl(Pd!31,l!32,r!29),Cl(Pp!7,d!0,l!30,r!32),Cl(
    Pp!13,d!9,l!33,r!34),Cl(Pd!14,Pp!17,d!20,l!35,r!33)
    ,Cl(Pd!18,Pp,d,l!34,r!35),Cl(Pp!27,d!31,l!36,r!37),Cl
    (Pd!28,Pp!39,d!42,l!38,r!36),Cl(Pd!40,l!37,r!38) @ 0(
```

```
'pring')

'pentagon␣closure' Cl(Pd!0,l!1,r!2),Cl(Pp!3,d!4,l!5,r!1)
    ,Cl(Pd!6,Pp!7,d!8,l!2,r!5),Cl(Pd!9,l!10,r!11),Cl(
    Pp,d,l!12,r!10),Cl(Pd,Pp!13,d!14,l!11,r!12),Cl(Pd!4,l
    !15,r!16),Cl(Pp!17,d!18,l!19,r!15),Cl(Pd!20,Pp!3,d!6
    ,l!16,r!19),Cl(Pd!8,Pp,d,l!29,r!30),Cl(Pd,l!32,r!29)
    ,Cl(Pp!7,d!0,l!30,r!32),Cl(Pp!13,d!9,l!33,r!34),Cl(Pd
    !14,Pp!17,d!20,l!35,r!33),Cl(Pd!18,l!34,r!35)->Cl(Pd
    !0,l!1,r!2),Cl(Pp!3,d!4,l!5,r!1),Cl(Pd!6,Pp!7,d!8,l!2
    ,r!5),Cl(Pd!9,l!10,r!11),Cl(Pp!36,d!38,l!12,r!10),Cl(
    Pd!37,Pp!13,d!14,l!11,r!12),Cl(Pd!4,l!15,r!16),Cl(Pp
    !17,d!18,l!19,r!15),Cl(Pd!20,Pp!3,d!6,l!16,r!19),Cl(
    Pd!8,Pp!36,d!37,l!29,r!30),Cl(Pd!38,l!32,r!29),Cl(Pp
    !7,d!0,l!30,r!32),Cl(Pp!13,d!9,l!33,r!34),Cl(Pd!14,Pp
    !17,d!20,l!35,r!33),Cl(Pd!18,l!34,r!35) @ 0('pring')
```

Adding regulatory mechanisms to the model

Code 12: Modification to take adaptor binding as a regulatory mechanism

```
%var: 'pringA6' 'pring'*10
%var: 'pringA5' 'pring'/10

'adaptor␣hexagon␣closure' Cl(Pd!0,l!1,r!2), Cl(Pp!3,d!4
    ,l!5,r!1), Cl(Pd!6,Pp!7,d!8,l!2,r!5), Cl(Pd!9,l!10,r
    !11), Cl(Pp,d,l!12,r!10), Cl(Pd,Pp!13,d!14,l!11,r!12),
     Cl(Pd!4,l!15,r!16), Cl(Pp!17,d!18,l!19,r!15), Cl(Pd
    !20,Pp!3,d!6,l!16,r!19), Cl(Pd!8,Pp!27,d!28,l!29,r!30)
    , Cl(Pd!31,l!32,r!29), Cl(Pp!7,d!0,l!30,r!32), Cl(Pp
    !13,d!9,l!33,r!34), Cl(Pd!14,Pp!17,d!20,l!35,r!33), Cl
    (Pd!18,l!34,r!35), Cl(Pp!27,d!31,l!36,r!37), Cl(Pd!28
    ,Pp,d,l!38,r!36,A!_), Cl(Pd,l!37,r!38)->Cl(Pd!0,l!1,r
    !2), Cl(Pp!3,d!4,l!5,r!1), Cl(Pd!6,Pp!7,d!8,l!2,r!5),
    Cl(Pd!9,l!10,r!11), Cl(Pp!39,d!40,l!12,r!10), Cl(Pd!42
    ,Pp!13,d!14,l!11,r!12), Cl(Pd!4,l!15,r!16), Cl(Pp!17,d
    !18,l!19,r!15), Cl(Pd!20,Pp!3,d!6,l!16,r!19), Cl(Pd!8
    ,Pp!27,d!28,l!29,r!30), Cl(Pd!31,l!32,r!29), Cl(Pp!7,d
    !0,l!30,r!32), Cl(Pp!13,d!9,l!33,r!34), Cl(Pd!14,Pp!17
    ,d!20,l!35,r!33), Cl(Pd!18,l!34,r!35), Cl(Pp!27,d!31,l
    !36,r!37), Cl(Pd!28,Pp!39,d!42,l!38,r!36,A!_), Cl(Pd
    !40,l!37,r!38) @ 0('pringA6')

'adaptor␣pentagon␣closure' Cl(Pd!0,l!1,r!2),Cl(Pp!3,d!4
    ,l!5,r!1),Cl(Pd!6,Pp!7,d!8,l!2,r!5),Cl(Pd!9,l!10,r
```

```
!11),Cl(Pp,d,l!12,r!10,A!_),Cl(Pd,Pp!13,d!14,l!11,r
!12),Cl(Pd!4,l!15,r!16),Cl(Pp!17,d!18,l!19,r!15),Cl(
Pd!20,Pp!3,d!6,l!16,r!19),Cl(Pd!8,Pp,d,l!29,r!30),Cl(
Pd,l!32,r!29),Cl(Pp!7,d!0,l!30,r!32),Cl(Pp!13,d!9,l
!33,r!34),Cl(Pd!14,Pp!17,d!20,l!35,r!33),Cl(Pd!18,l
!34,r!35)->Cl(Pd!0,l!1,r!2),Cl(Pp!3,d!4,l!5,r!1),Cl(
Pd!6,Pp!7,d!8,l!2,r!5),Cl(Pd!9,l!10,r!11),Cl(Pp!36,d
!38,l!12,r!10,A!_),Cl(Pd!37,Pp!13,d!14,l!11,r!12),Cl(
Pd!4,l!15,r!16),Cl(Pp!17,d!18,l!19,r!15),Cl(Pd!20,Pp
!3,d!6,l!16,r!19),Cl(Pd!8,Pp!36,d!37,l!29,r!30),Cl(Pd
!38,l!32,r!29),Cl(Pp!7,d!0,l!30,r!32),Cl(Pp!13,d!9,l
!33,r!34),Cl(Pd!14,Pp!17,d!20,l!35,r!33),Cl(Pd!18,l
!34,r!35) @ 0('pringA5')
```

Code 13: Modification to take serine phosphorylation as a regulatory mechanism

```
%var: 'pringP6' 'pring'*10
%var: 'pringP5' 'pring'/10

'phosphorylation␣hexagon␣closure' Cl(Pd!0,l!1,r!2), Cl(
  Pp!3,d!4,l!5,r!1), Cl(Pd!6,Pp!7,d!8,l!2,r!5), Cl(Pd!9
  ,l!10,r!11), Cl(Pp,d,l!12,r!10), Cl(Pd,Pp!13,d!14,l!11
  ,r!12), Cl(Pd!4,l!15,r!16), Cl(Pp!17,d!18,l!19,r!15),
  Cl(Pd!20,Pp!3,d!6,l!16,r!19), Cl(Pd!8,Pp!27,d!28,l!29
  ,r!30), Cl(Pd!31,l!32,r!29), Cl(Pp!7,d!0,l!30,r!32),
  Cl(Pp!13,d!9,l!33,r!34), Cl(Pd!14,Pp!17,d!20,l!35,r
  !33), Cl(Pd!18,l!34,r!35), Cl(Pp!27,d!31,l!36,r!37),
  Cl(Pd!28,Pp,d,l!38,r!36,S~P), Cl(Pd,l!37,r!38)->Cl(Pd
  !0,l!1,r!2), Cl(Pp!3,d!4,l!5,r!1), Cl(Pd!6,Pp!7,d!8,l
  !2,r!5), Cl(Pd!9,l!10,r!11), Cl(Pp!39,d!40,l!12,r!10),
   Cl(Pd!42,Pp!13,d!14,l!11,r!12), Cl(Pd!4,l!15,r!16),
  Cl(Pp!17,d!18,l!19,r!15), Cl(Pd!20,Pp!3,d!6,l!16,r!19)
  , Cl(Pd!8,Pp!27,d!28,l!29,r!30), Cl(Pd!31,l!32,r!29),
  Cl(Pp!7,d!0,l!30,r!32), Cl(Pp!13,d!9,l!33,r!34), Cl(Pd
  !14,Pp!17,d!20,l!35,r!33), Cl(Pd!18,l!34,r!35), Cl(Pp
  !27,d!31,l!36,r!37), Cl(Pd!28,Pp!39,d!42,l!38,r!36,S~P
  ), Cl(Pd!40,l!37,r!38) @ 0('pringP6')

'posphorylation␣pentagon␣closure' Cl(Pd!0,l!1,r!2),Cl(Pp
   !3,d!4,l!5,r!1),Cl(Pd!6,Pp!7,d!8,l!2,r!5),Cl(Pd!9,l
   !10,r!11),Cl(Pp,d,l!12,r!10,S~P),Cl(Pd,Pp!13,d!14,l
   !11,r!12),Cl(Pd!4,l!15,r!16),Cl(Pp!17,d!18,l!19,r!15)
   ,Cl(Pd!20,Pp!3,d!6,l!16,r!19),Cl(Pd!8,Pp,d,l!29,r!30)
   ,Cl(Pd,l!32,r!29),Cl(Pp!7,d!0,l!30,r!32),Cl(Pp!13,d!9
   ,l!33,r!34),Cl(Pd!14,Pp!17,d!20,l!35,r!33),Cl(Pd!18,l
   !34,r!35)->Cl(Pd!0,l!1,r!2),Cl(Pp!3,d!4,l!5,r!1),Cl(
```

```
Pd!6,Pp!7,d!8,l!2,r!5),Cl(Pd!9,l!10,r!11),Cl(Pp!36,d
!38,l!12,r!10,S~P),Cl(Pd!37,Pp!13,d!14,l!11,r!12),Cl(
Pd!4,l!15,r!16),Cl(Pp!17,d!18,l!19,r!15),Cl(Pd!20,Pp
!3,d!6,l!16,r!19),Cl(Pd!8,Pp!36,d!37,l!29,r!30),Cl(Pd
!38,l!32,r!29),Cl(Pp!7,d!0,l!30,r!32),Cl(Pp!13,d!9,l
!33,r!34),Cl(Pd!14,Pp!17,d!20,l!35,r!33),Cl(Pd!18,l
!34,r!35) @ 0('pringP5')
```

Probability to close the ring

To calculate ratio of probabilities of closing a ring with l bonds and $k < l$ bonds, assume we have a complex of n bonds already. There is C_n^l possible combinations of l bonds for larger ring to close, and there is C_n^k possible combinations of k bonds for smaller ring to close. Ratio of that numbers gives us ratio of larger rings closing rate to smaller ring closing rate. Assume that $l = k + m$:

$$\alpha = \frac{C_n^l}{C_n^k} = \frac{n!}{(n-l)!l!}\frac{(n-k)!k!}{n!} = \frac{(n-k)!}{(n-l)!}\frac{k!}{l!} = \\ = \frac{\prod_{i=1}^{m} n-l-i}{\prod_{i=1}^{m} k+i} = \prod_{i=1}^{m}\frac{n-l-i}{k+i} = \prod_{i=1}^{m}\alpha_i \geq \left(\frac{n-k}{l}\right)^{(l-k)} \quad (10)$$

In the last product all $\alpha_i > 1$, if the larger ring length $l < n/2$, so all product is greater than unity and growth quite fast with l. This formula explains why we haven't observe rings of length 5 and 6 even in the Model IIIb.

$$K = \frac{k_+}{k_-} = \frac{pbk \cdot (N_A \cdot V)}{pdk} \quad (11)$$

$$\Delta G = -RT \ln K \quad (12)$$

References

1. Perelson, A. S., and B. Goldstein, 1985. The equilibrium aggregate size distribution of self-associating trivalent molecules. *Macromolecules* .

2. Danos, V., J. Feret, W. Fontana, R. Harmer, and J. Krivine, 2009. Rule-based modelling and model perturbation. *Transactions on Computational Systems Biology XI* 116–137.

3. metakappa. https://github.com/kappamodeler/metakappa.

4. den Otter, W. K., M. R. Renes, and W. J. Briels, 2010. Asymmetry as the Key to Clathrin Cage Assembly. *Biophys J* 99:1231–1238.

5. Fotin, A., Y. Cheng, P. Sliz, N. Grigorieff, S. C. Harrison, T. Kirchhausen, and T. Walz, 2004. Molecular model for a complete clathrin lattice from electron cryomicroscopy. *Nature* 432:573–579.

6. Ferguson, M. L., K. Prasad, H. Boukari, D. L. Sackett, S. Krueger, E. M. Lafer, and R. Nossal, 2008. Clathrin triskelia show evidence of molecular flexibility. *Biophys J* 95:1945–1955.

Additional figures

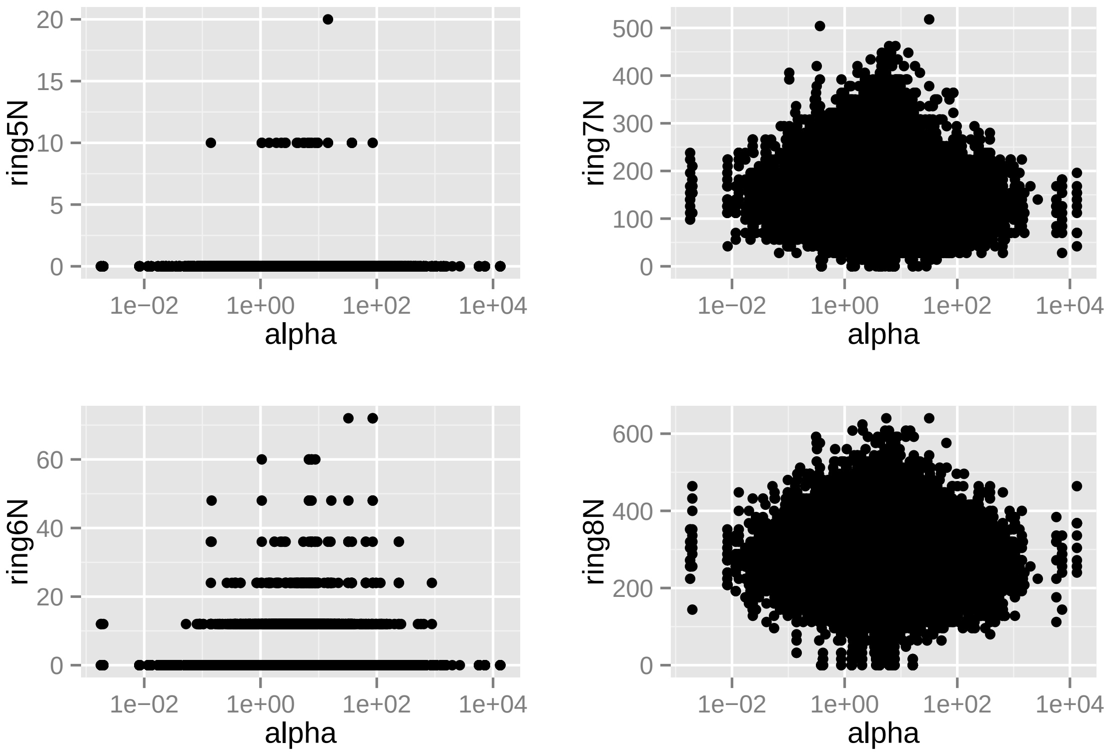

Supplementary Figure 3: Number of rings formed in Model II. It could be seen that the number of rings is growing with the ring size as explained in the section “Probability to close the ring” on the page 13.

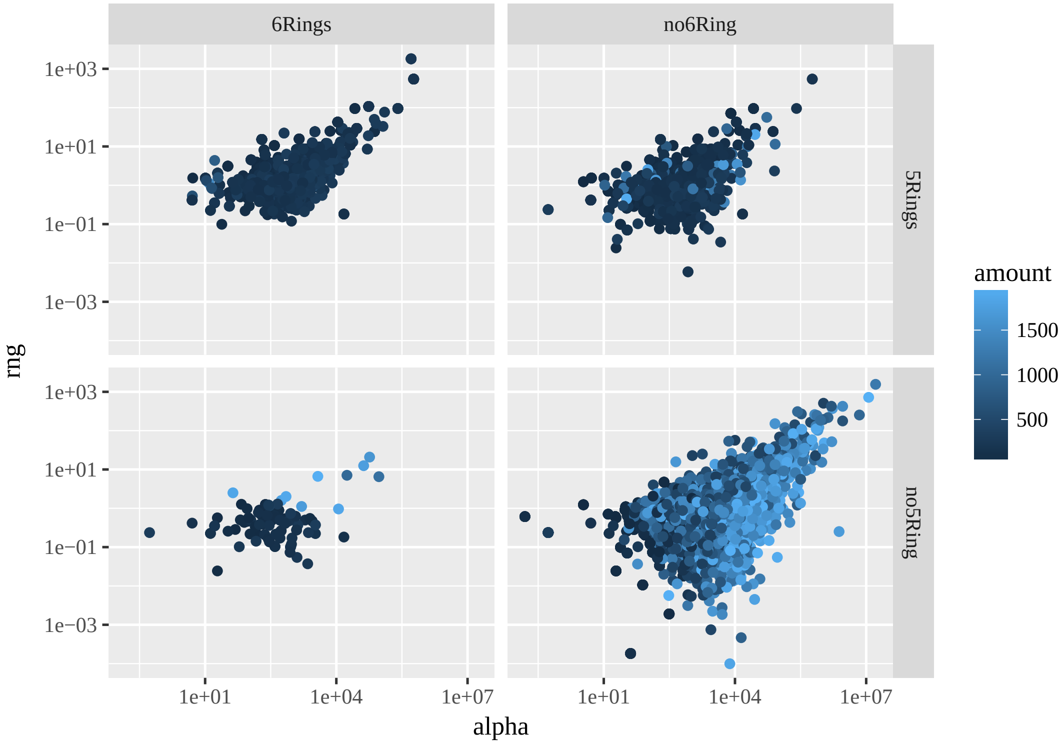

Supplementary Figure 4: Distribution of alpha and rng parameters. Parameter “rng” is the ratio of ring closure and ring break rate constants. Parameter “alpha” is dimensionless monomer association equilibrium constant. Here we have plot all combinations of “alpha” and “rng” parameters split into four panels by the presence/absence of pentagons and hexagons. First observation is that there is no clear separation between presence and absence datasets. It is also interesting that the vast majority of datasets located in right bottom panel (no5Ring/no6Ring) and produce no rings at all.

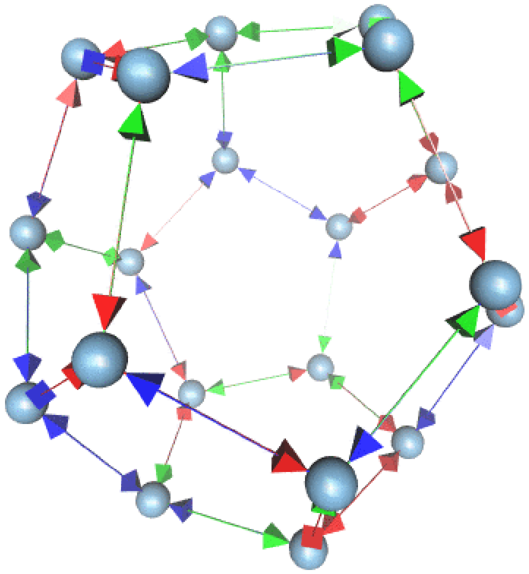

Supplementary Figure 5: Dodecahedron structure obtained by simulation of the unconstrained model from the mixture of free triskelia.

Supplementary Table 1: Sensitivity coefficients for Model II

	pbk	pv.pbk	pdk	pv.pdk	pring	pv.pring	amount	pv.amount
Ncplx	-8.358E-03	5.551E-01	2.490E-02	7.875E-02	-2.218E-02	1.173E-01	8.798E-01	0.000E+00
Nstruc	-2.850E-02	4.422E-02	3.444E-02	1.502E-02	-1.288E-02	3.633E-01	9.300E-01	0.000E+00
Wmax	-1.776E-03	9.002E-01	-7.351E-04	9.586E-01	1.756E-02	2.151E-01	1.066E-01	4.552E-14
nWmax	3.960E-03	7.798E-01	1.309E-03	9.264E-01	-2.742E-02	5.286E-02	-1.657E-01	0.000E+00
Nmax	-2.793E-03	8.437E-01	1.580E-02	2.647E-01	-1.969E-02	1.646E-01	8.479E-01	0.000E+00
wNmax	1.391E-02	3.261E-01	-6.561E-02	3.546E-06	1.711E-02	2.272E-01	-5.379E-01	0.000E+00
Nfree	-8.591E-04	9.516E-01	1.995E-02	1.590E-01	-2.044E-02	1.490E-01	8.483E-01	0.000E+00
Rext	-4.497E-03	7.509E-01	-1.629E-02	2.501E-01	2.691E-02	5.742E-02	-3.992E-01	0.000E+00
Crank	-4.969E-02	4.483E-04	-4.831E-02	6.435E-04	8.583E-02	1.279E-09	-5.843E-01	0.000E+00
ring6N	-1.855E-02	1.904E-01	-7.138E-02	4.528E-07	1.033E-01	2.671E-13	-5.174E-01	0.000E+00
ring5N	-4.624E-02	1.090E-03	-4.935E-02	4.900E-04	8.542E-02	1.529E-09	-5.866E-01	0.000E+00
ring7N	-9.088E-03	5.211E-01	-4.005E-02	4.675E-03	1.535E-02	2.786E-01	-3.657E-02	9.807E-03
ring8N	-1.598E-02	2.592E-01	-9.942E-02	1.983E-12	1.235E-01	0.000E+00	-5.011E-01	0.000E+00
g501	1.017E-03	9.428E-01	-1.184E-02	4.034E-01	-3.946E-03	7.806E-01	2.120E-02	1.344E-01
g511	1.017E-03	9.428E-01	-1.184E-02	4.034E-01	-3.946E-03	7.806E-01	2.120E-02	1.344E-01
g521	1.017E-03	9.428E-01	-1.184E-02	4.034E-01	-3.946E-03	7.806E-01	2.120E-02	1.344E-01
g522	1.017E-03	9.428E-01	-1.184E-02	4.034E-01	-3.946E-03	7.806E-01	2.120E-02	1.344E-01
g531	-1.414E-02	3.180E-01	-9.265E-02	5.582E-11	4.859E-02	5.981E-04	-1.014E-01	7.061E-13
g532	-1.965E-02	1.654E-01	-7.368E-02	1.905E-07	3.773E-02	7.716E-03	-8.541E-02	1.537E-09
g541	-9.455E-03	5.045E-01	-1.728E-01	0.000E+00	9.076E-02	1.360E-10	-2.051E-01	0.000E+00
g551	-2.150E-02	1.290E-01	-1.931E-01	0.000E+00	9.078E-02	1.348E-10	-2.347E-01	0.000E+00
g601	1.017E-03	9.428E-01	-1.184E-02	4.034E-01	-3.946E-03	7.806E-01	2.120E-02	1.344E-01
g611	1.017E-03	9.428E-01	-1.184E-02	4.034E-01	-3.946E-03	7.806E-01	2.120E-02	1.344E-01
g621	1.017E-03	9.428E-01	-1.184E-02	4.034E-01	-3.946E-03	7.806E-01	2.120E-02	1.344E-01
g622	1.017E-03	9.428E-01	-1.184E-02	4.034E-01	-3.946E-03	7.806E-01	2.120E-02	1.344E-01
g623	1.017E-03	9.428E-01	-1.184E-02	4.034E-01	-3.946E-03	7.806E-01	2.120E-02	1.344E-01
g631	1.017E-03	9.428E-01	-1.184E-02	4.034E-01	-3.946E-03	7.806E-01	2.120E-02	1.344E-01
g632	1.017E-03	9.428E-01	-1.184E-02	4.034E-01	-3.946E-03	7.806E-01	2.120E-02	1.344E-01
g633	1.017E-03	9.428E-01	-1.184E-02	4.034E-01	-3.946E-03	7.806E-01	2.120E-02	1.344E-01
g641	-4.420E-03	7.550E-01	-3.310E-02	1.942E-02	-2.647E-03	8.518E-01	-2.934E-02	3.830E-02
g642	1.017E-03	9.428E-01	-1.184E-02	4.034E-01	-3.946E-03	7.806E-01	2.120E-02	1.344E-01
g643	3.963E-03	7.796E-01	-2.452E-02	8.337E-02	2.562E-03	8.565E-01	-2.082E-02	1.416E-01
g651	-2.044E-02	1.490E-01	-7.994E-02	1.583E-08	3.797E-02	7.324E-03	-8.931E-02	2.664E-10
g661	-3.152E-04	9.822E-01	-1.227E-01	0.000E+00	3.924E-02	5.588E-03	-1.262E-01	0.000E+00

Supplementary Table 2: Sensitivity coefficients for the budding from flat raft in the Model II

	b	pval.b	d	pval.d	rng5	pval.rng5	rng6	pval.rng6
Ncplx	-1.501E-02	1.545E-01	-1.775E-02	9.224E-02	1.596E-02	1.300E-01	7.285E-03	4.896E-01
Nstruc	1.597E-02	1.298E-01	1.615E-02	1.256E-01	-4.711E-03	6.550E-01	-1.070E-02	3.101E-01
Wmax	8.353E-03	4.282E-01	1.690E-02	1.089E-01	-1.795E-02	8.865E-02	2.481E-03	8.140E-01
nWmax	3.211E-03	7.607E-01	4.462E-03	6.722E-01	-3.254E-03	7.576E-01	-8.094E-04	9.388E-01
Nmax	-1.584E-02	1.329E-01	-1.803E-02	8.725E-02	1.591E-02	1.312E-01	8.422E-03	4.244E-01
wNmax	1.071E-02	3.100E-01	-5.525E-01	0.000E+00	-1.022E-02	3.324E-01	-5.583E-01	0.000E+00
Nfree	-1.584E-02	1.329E-01	-1.803E-02	8.725E-02	1.591E-02	1.312E-01	8.422E-03	4.244E-01
Rext	1.401E-02	1.839E-01	1.880E-02	7.457E-02	-1.647E-02	1.183E-01	-4.585E-03	6.637E-01
Crank	1.041E-02	3.235E-01	1.745E-02	9.784E-02	-1.687E-02	1.095E-01	-6.966E-06	9.995E-01
ring6N	7.544E-03	4.743E-01	1.073E-02	3.088E-01	-7.695E-03	4.655E-01	-2.059E-03	8.452E-01
ring5N	9.740E-03	3.556E-01	1.664E-02	1.146E-01	-1.673E-02	1.126E-01	2.874E-04	9.783E-01
ring7N	2.916E-03	7.821E-01	3.940E-03	7.087E-01	-2.888E-03	7.841E-01	-7.407E-04	9.440E-01
ring8N	3.939E-03	7.087E-01	1.187E-02	2.604E-01	-1.491E-02	1.572E-01	5.000E-03	6.353E-01
g501	1.071E-02	3.100E-01	-5.525E-01	0.000E+00	-1.022E-02	3.324E-01	-5.583E-01	0.000E+00
g511	1.071E-02	3.100E-01	-5.525E-01	0.000E+00	-1.022E-02	3.324E-01	-5.583E-01	0.000E+00
g521	1.071E-02	3.100E-01	-5.525E-01	0.000E+00	-1.022E-02	3.324E-01	-5.583E-01	0.000E+00
g522	1.071E-02	3.100E-01	-5.525E-01	0.000E+00	-1.022E-02	3.324E-01	-5.583E-01	0.000E+00
g531	2.087E-04	9.842E-01	1.929E-04	9.854E-01	-4.123E-05	9.969E-01	-4.373E-05	9.967E-01
g532	8.246E-04	9.377E-01	1.221E-03	9.078E-01	-6.304E-04	9.523E-01	-1.984E-04	9.850E-01
g541	3.819E-03	7.172E-01	5.417E-03	6.074E-01	-3.834E-03	7.161E-01	-1.029E-03	9.222E-01
g551	-1.249E-02	2.362E-01	-1.440E-03	8.914E-01	-1.592E-02	1.312E-01	1.936E-02	6.634E-02
g601	1.071E-02	3.100E-01	-5.525E-01	0.000E+00	-1.022E-02	3.324E-01	-5.583E-01	0.000E+00
g611	1.071E-02	3.100E-01	-5.525E-01	0.000E+00	-1.022E-02	3.324E-01	-5.583E-01	0.000E+00
g621	1.071E-02	3.100E-01	-5.525E-01	0.000E+00	-1.022E-02	3.324E-01	-5.583E-01	0.000E+00
g622	1.071E-02	3.100E-01	-5.525E-01	0.000E+00	-1.022E-02	3.324E-01	-5.583E-01	0.000E+00
g623	1.071E-02	3.100E-01	-5.525E-01	0.000E+00	-1.022E-02	3.324E-01	-5.583E-01	0.000E+00
g631	1.071E-02	3.100E-01	-5.525E-01	0.000E+00	-1.022E-02	3.324E-01	-5.583E-01	0.000E+00
g632	1.071E-02	3.100E-01	-5.525E-01	0.000E+00	-1.022E-02	3.324E-01	-5.583E-01	0.000E+00
g633	1.071E-02	3.100E-01	-5.525E-01	0.000E+00	-1.022E-02	3.324E-01	-5.583E-01	0.000E+00
g641	1.071E-02	3.100E-01	-5.525E-01	0.000E+00	-1.022E-02	3.324E-01	-5.583E-01	0.000E+00
g642	1.071E-02	3.100E-01	-5.525E-01	0.000E+00	-1.022E-02	3.324E-01	-5.583E-01	0.000E+00
g643	1.071E-02	3.100E-01	-5.525E-01	0.000E+00	-1.022E-02	3.324E-01	-5.583E-01	0.000E+00
g651	2.087E-04	9.842E-01	1.929E-04	9.854E-01	-4.123E-05	9.969E-01	-4.373E-05	9.967E-01
g661	3.744E-03	7.225E-01	5.277E-03	6.167E-01	-3.707E-03	7.252E-01	-1.029E-03	9.222E-01
