# Supplementary figures and images for: Rule-based modelling provides an extendable framework for comparing candidate mechanisms underpinning clathrin polymerisation
Source: Sci Rep. 2018 Apr 4;8:5658. doi: 10.1038/s41598-018-23829-x (PMC5884807; doi:10.1038/s41598-018-23829-x)

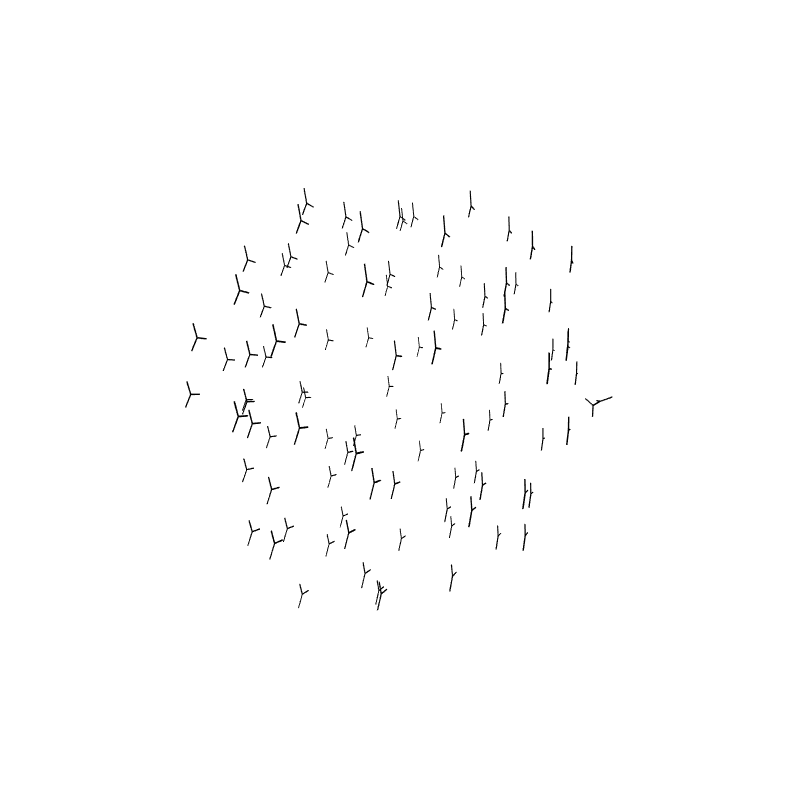

Supplement: Supplementary file 2 — Supplementary movie 1 [file 41598_2018_23829_MOESM2_ESM.gif]

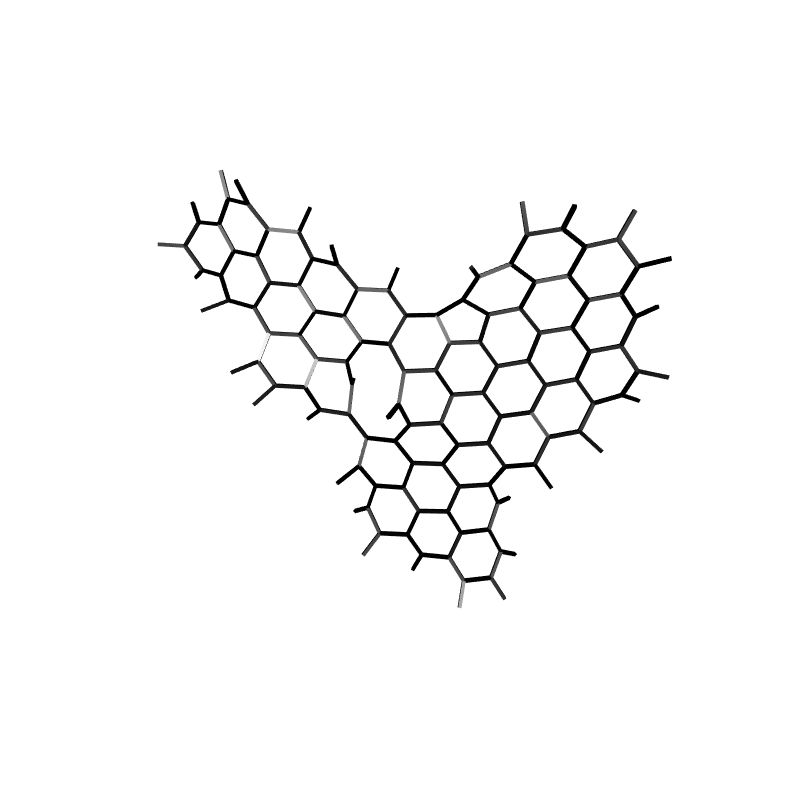

Supplement: Supplementary file 3 — Supplementary movie 2 [file 41598_2018_23829_MOESM3_ESM.gif]

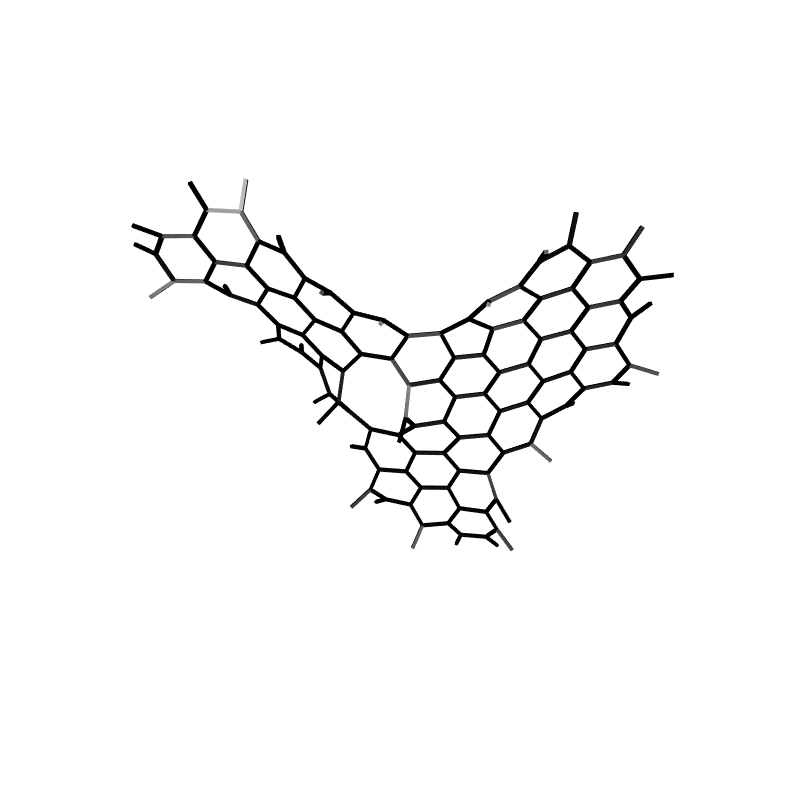

Supplement: Supplementary file 4 — Supplementary movie 3 [file 41598_2018_23829_MOESM4_ESM.gif]
